# Supplementary material for: DNA methylation profiling enables subclassification of mucinous ovarian carcinoma and distinguishes it from extraovarian mucinous metastases
Source: Cell Rep Med. 2026 Jul 27;7(8):102941. doi: 10.1016/j.xcrm.2026.102941 (PMC13522789; doi:10.1016/j.xcrm.2026.102941)
Supplement: Supplementary file 1 [file mmc1.pdf]

Cell Reports Medicine, Volume *7*

Supplemental information

DNA methylation profiling enables subclassification of mucinous ovarian carcinoma and distinguishes it from extraovarian mucinous metastases

Teodor G. Calina, Elena Grafenhorst, Simon Schallenberg, Markus Möbs, Bjarne Daenekas, Alexandru A. Sabo, BaoQing Chen, Ines Koch, Tobias Janik, Michael Churchman, Charlie Gourley, Elizabeth L. Christie, Catalin Vasilescu, Vlad Herlea, Wolfgang D. Schmitt, Nicolae Leopold, Florian Heitz, Angelina Vlaski, Yuri Tolkach, Felix Bremmer, Frederick Klauschen, Reinhard Büttner, Philipp Ströbel, Eike Burandt, Guido Sauter, Frank L. Heppner, Eliane T. Taube, Jalid Sehouli, Ioana E. Braicu, Philipp Euskirchen, David Horst, Kylie L. Gorringe, David Capper, and Mihnea P. Dragomir

Supplementary Figures

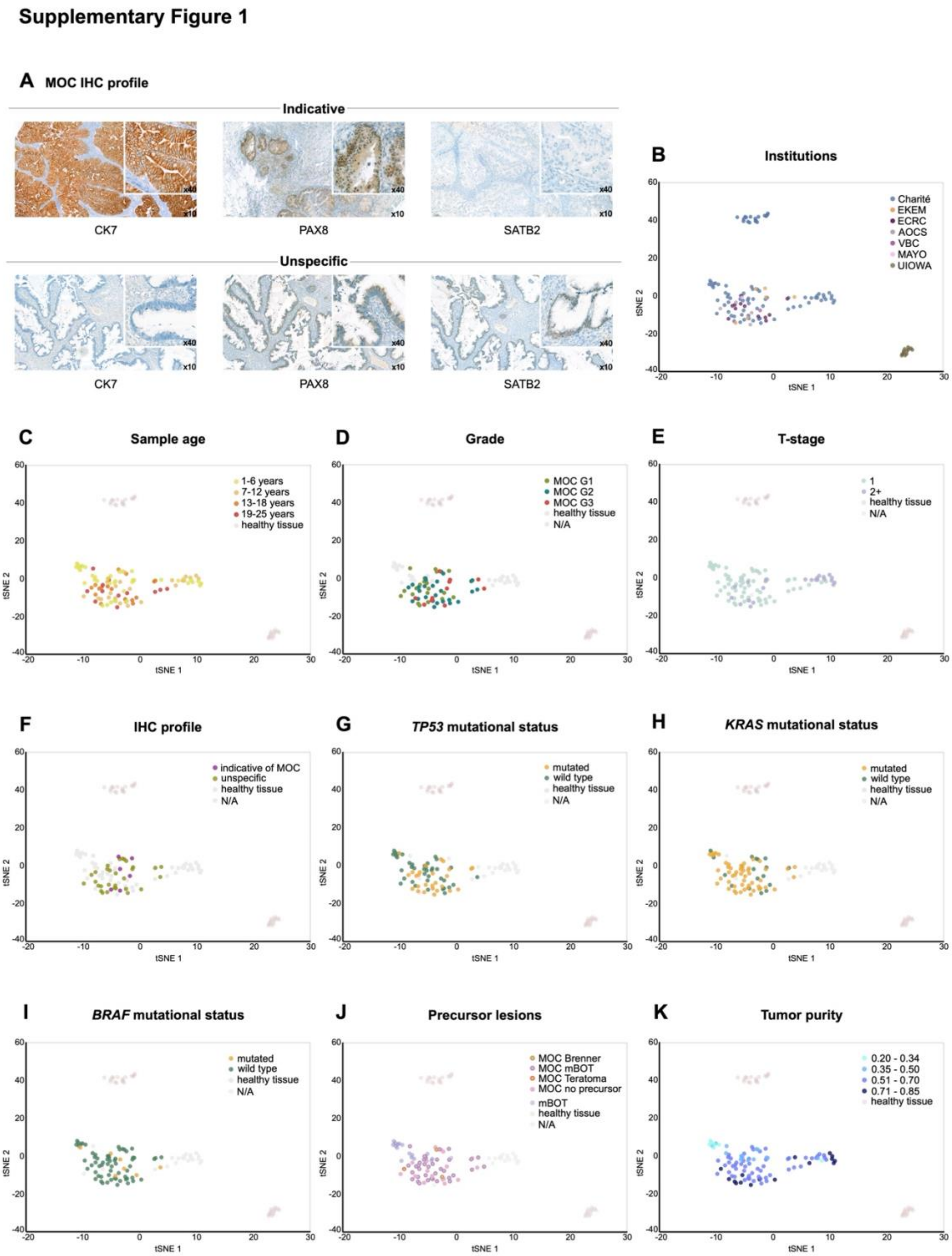

Figure S1. Characterization of the mucinous ovarian carcinoma (MOC) cohort. Related to Figures 1.
(A) Representative IHC images of CK7, PAX8, and SATB2 staining in MOC with indicative (upper panel) or unspecific IHC profiles (lower panel).
(B–K) t-SNE plots of MOC and other ovarian lesions and normal tissues colored by (B) institution, (C) sample age, (D) grade, (E) T-stage, (F) IHC profile, (G) *TP53* mutational status, (H) *KRAS* mutational status, (I) *BRAF*

mutational status, (J) precursor lesions (Brenner tumor, mBOT, teratoma, no precursor, and N/A), and (K) tumor purity.

Supplementary Figure 2

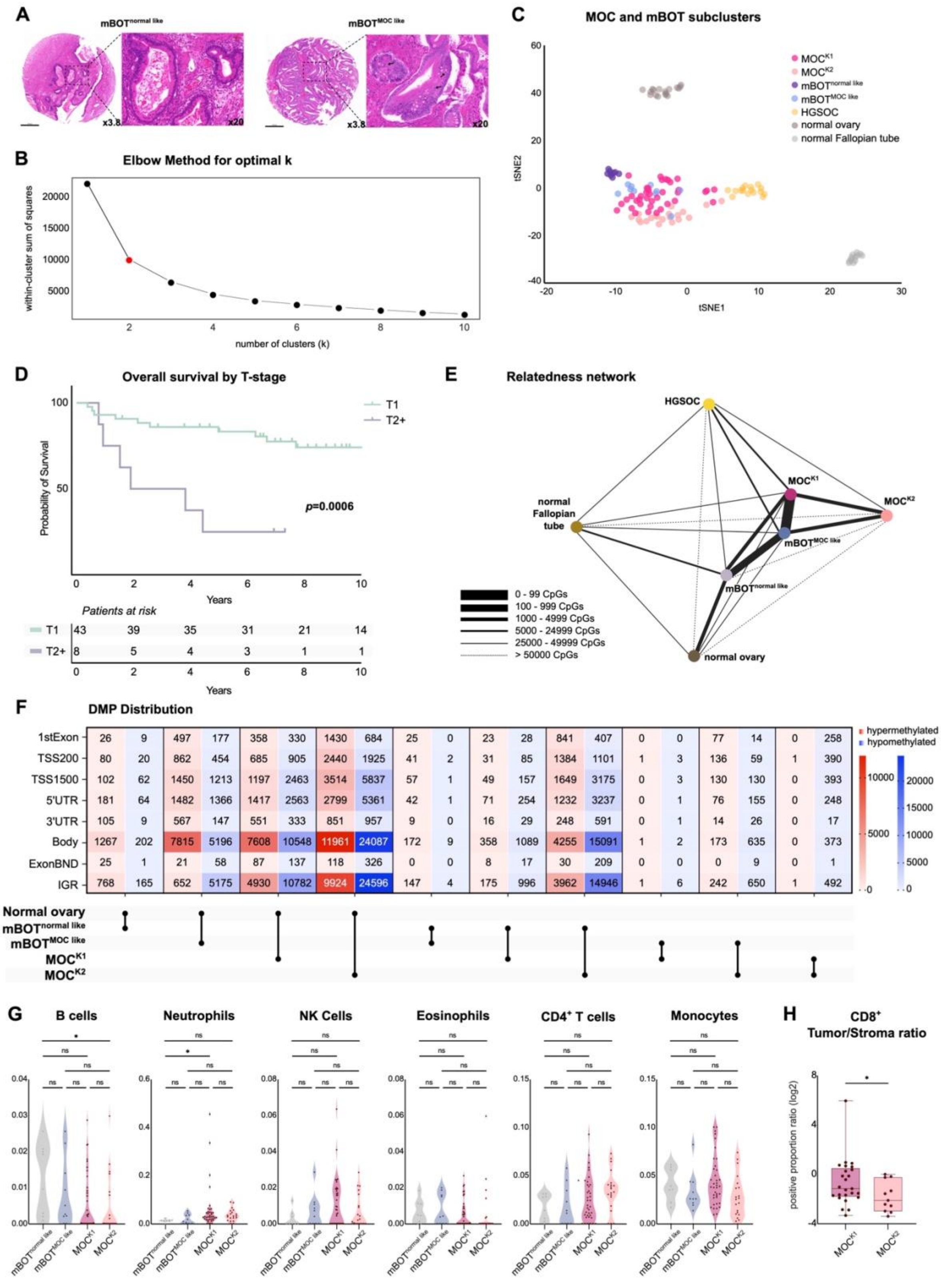

Figure S2. Characterization of mBOT and MOC subtypes. Related to Figures 2.

(A) Representative H&E images of mBOT subtypes at low (×3.8) and high (×20) magnification. Arrows marking mitoses. Scale bars, 500 µm.

(B) Elbow plot of within-cluster sum of squares used to determine the optimal k-means cluster number.

(C) t-SNE plot (same as in Fig. 1D) highlighting $mBOT^{normal\_like}$, $mBOT^{MOC\_like}$, MOC^{K1}, and MOC^{K2} subclusters, along with normal ovary, Fallopian tube, and HGSOC.
(D) Kaplan–Meier survival of MOC patients stratified by T-stage. *P* value from Mantel–Cox log-rank test.
(E) Relatedness network of methylation subtypes showing connections among $mBOT^{normal\_like}$, $mBOT^{MOC\_like}$, MOC^{K1}, MOC^{K2}, normal ovary, normal Fallopian tube, and HGSOC. Node size reflects group size, and edge thickness indicates the number of differentially methylated CpGs.
(F) Heatmap of differentially methylated probes (DMPs) across genomic features. Color intensity represents frequency of hypermethylated (red) and hypomethylated (blue) probes.
(G) Estimated immune cell proportions (y axis, % of positive cells) for B cells, neutrophils, NK cells, eosinophils, $CD4^+$ T cells, and monocytes in mBOT and MOC subtypes. Comparisons by Kruskal–Wallis with Dunn's correction. ns, not significant; $*p < 0.05$.
(H) Tumor-to-stroma ratio of $CD8^+$ T cell positive proportion in MOC^{K1} and MOC^{K2}. Data are shown as log2-transformed ratios; statistical comparisons were made using Mann Whitney test with $*p < 0.05$. Positive values indicate relative enrichment of $CD8^+$ T cells within tumor regions, whereas negative values indicate relative enrichment within the stromal compartment.

Supplementary Figure 3

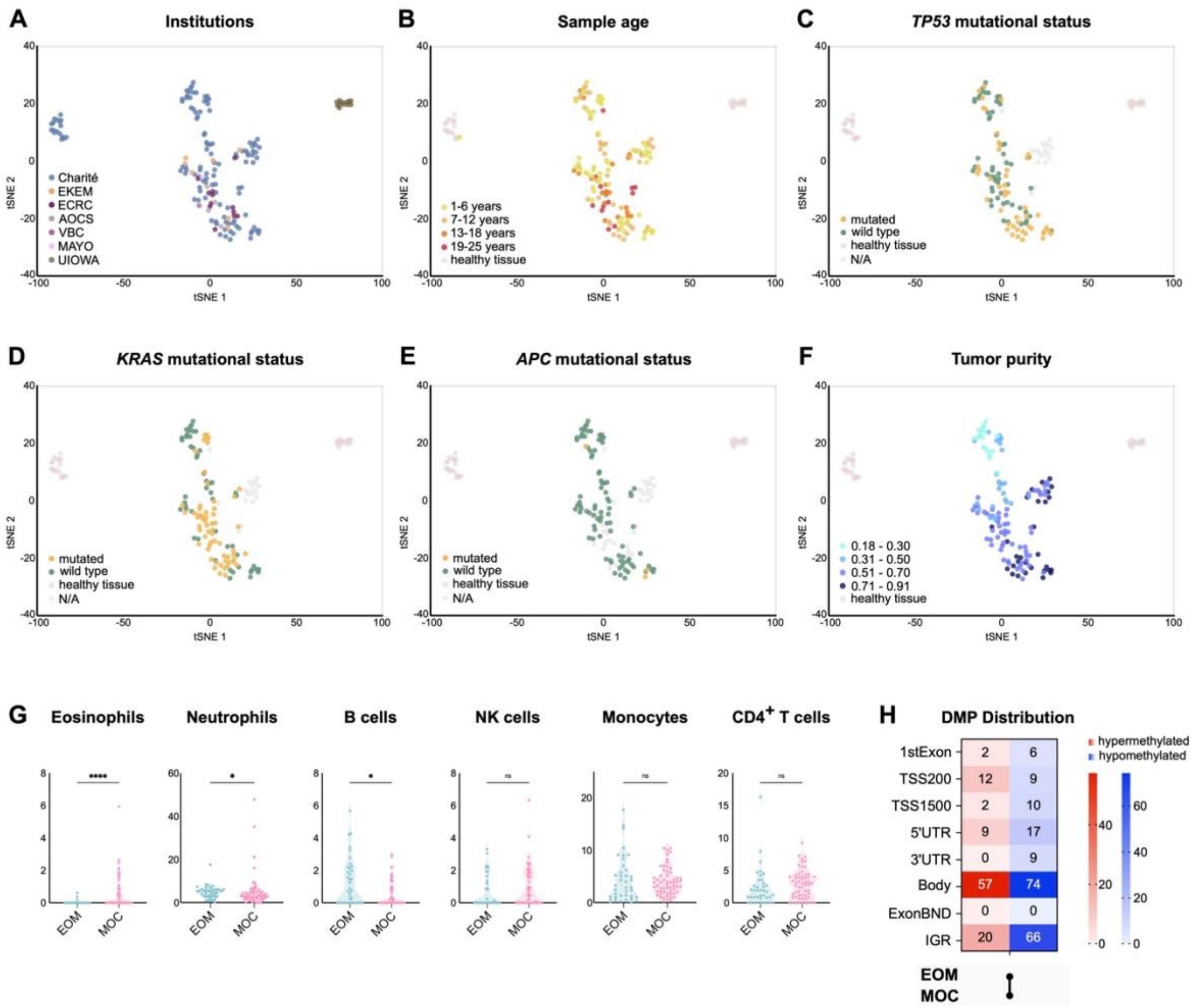

Figure S3. Characterization of the extra-ovarian metastasis (EOM) cohort. Related to Figures 3.
(A–F) t-SNE plots of MOC and related tissues colored by (A) institution, (B) sample age, (C) *TP53* mutational status, (D) *KRAS* mutational status, (E) *APC* mutational status, and (F) tumor purity.
(G) Estimated immune cell proportions (y axis, % of positive cells) for eosinophils, neutrophils, B cells, NK cells, monocytes, and CD4+ T cells in EOM and MOC. Comparisons by Kruskal–Wallis with Dunn's correction. ns, not significant; *p < 0.05; ****p < 0.0001
(H) Heatmap of DMP distribution across genomic features comparing EOM and MOC. Color intensity represents frequency of hypermethylated (red) and hypomethylated (blue) probes.

Supplementary Figure 4

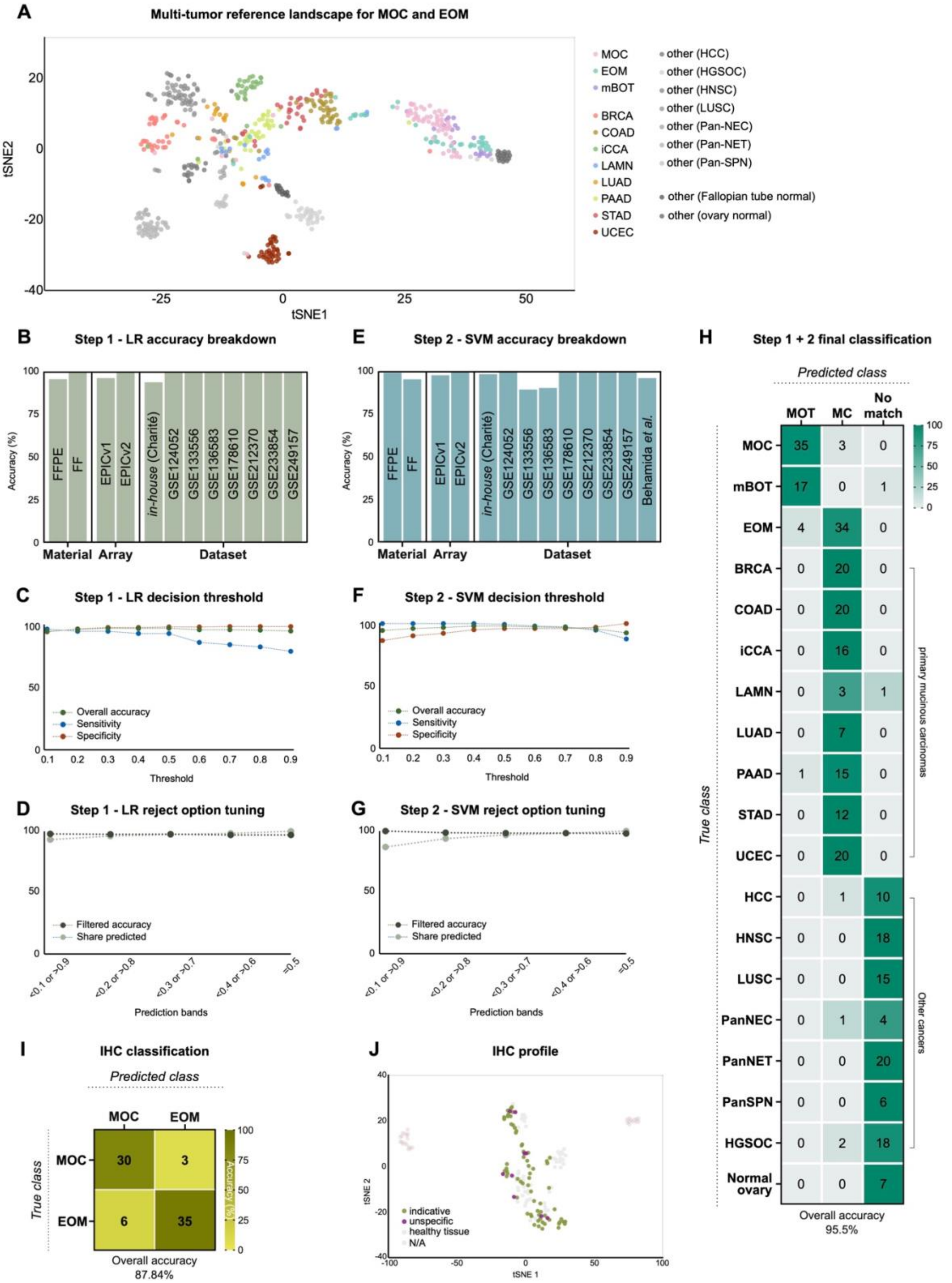

Figure S4. Performance of the step 1 and step 2 classifiers and of the IHC classifier. Related to Figures 4. (A) t-SNE visualization of the multi-tissue landscape used for classifier development and internal validation. (BRCA: breast adenocarcinoma; COAD: colorectal adenocarcinoma; iCCA: intrahepatic cholangiocarcinoma; LAMN: low-grade appendiceal mucinous neoplasm; LUAD: lung adenocarcinoma; PAAD: pancreatic

adenocarcinoma; STAD: stomach adenocarcinoma; UCEC: uterine corpus endometrial carcinoma; HCC: hepatocellular carcinoma; HGSOC: high-grade serous ovarian cancer; HNSC: head and neck squamous cell carcinoma; LUSC: lung squamous cell carcinoma; Pan-NEC: pancreatic neuroendocrine carcinoma; Pan-NET: pancreatic neuroendocrine tumor; Pan-SPN: pancreatic solid pseudopapillary neoplasms).
(B–D) Step 1 logistic regression (LR) classification: (B) accuracy across material type, array platform, and dataset. (*In-house (Charité)* refers to all samples available in-house, including those from the Charité, EKEM, and the PMCC collective); (C) performance across decision thresholds (0.1–0.9) for overall accuracy, sensitivity, and specificity; (D) reject-option analysis showing filtered accuracy and predicted share.
(E–G) Step 2 support vector machine (SVM) classification: (E) accuracy across material type, array platform, and dataset. (*In-house (Charité)* refers to all samples available in-house, including those from the Charité, EKEM, and the PMCC collective); (F) performance across thresholds; (G) reject-option analysis.
(H) Confusion matrix for combined two-step classification (Step 1 LR + Step 2 SVM) across all classes (overall accuracy: 95.5%).
(I) Confusion matrix for IHC-based classification of MOC versus EOM (overall accuracy: 87.8%).
(J) t-SNE plot (same as in Fig. 3C) showing samples classified as indicative versus unspecific MOC based on IHC profile.

Supplementary Figure 5

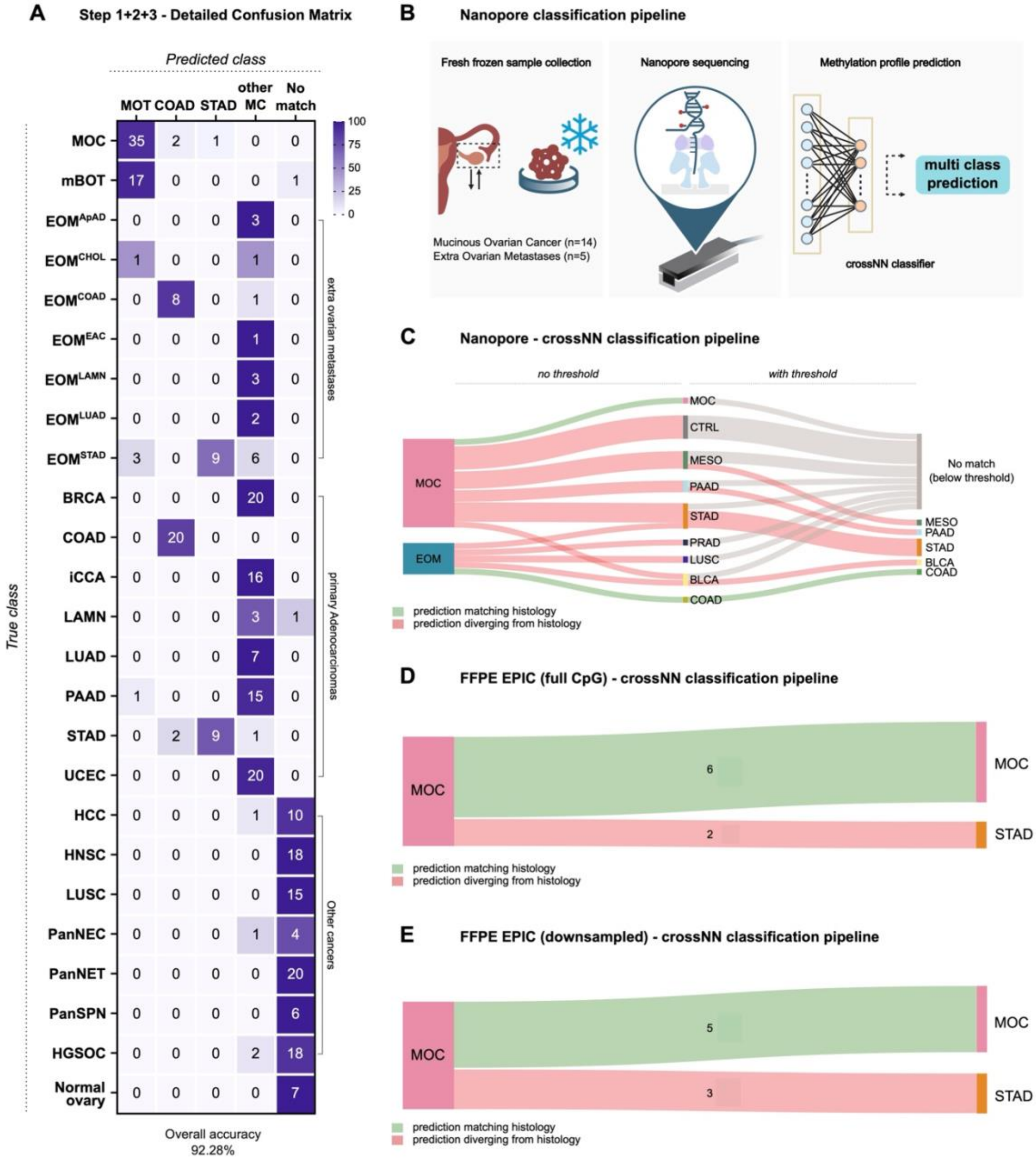

Figure S5. **Expanded classifier performance (Step 1+2+3), Nanopore diagnostic pipeline and results with and without CpG downsampling. Related to Figures 5.**

(A) Confusion matrix summarizing the final three-step DNA methylation classifier (Steps 1-3) across 25 histological classes/anatomical sites (overall accuracy: 92.28%).

(B) Schematic of the Nanopore-based classification workflow: fresh frozen samples undergo Nanopore sequencing, followed by methylation profile prediction and classification into MOT or EOM using the crossNN model. We deployed an expanded crossNN classifier version (trained on 20 MOC samples). Created with BioRender.com.

(C) Sankey plot showing per-sample Nanopore classification results using the crossNN classifier. Predictions are compared with histopathological diagnosis; green lines indicate concordance, and red lines indicate divergence. The classifier was tested on 19 intraoperative FF samples (14 MOC, 5 EOM). Results show that only 1/14 MOC

samples was correctly identified via Nanopore sequencing, with the majority of cases misclassified as non-MOC tumor types or healthy control tissue. Notably, one MOC sample containing Brenner tumor components was misclassified as bladder cancer (BLCA), likely due to shared urothelial differentiation signatures.
(D) Comparative Analysis of Matched EPIC Array and Nanopore Cohorts. To isolate the cause of low Nanopore accuracy, we analyzed a subset of 8 MOC cases profiled by both EPIC array (FFPE) and Nanopore (FF). When utilizing the full CpG set of the EPIC array, 6/8 samples were correctly classified as MOC. This indicates that the methylation signatures required for identification are present in the tumor tissue, though secondary misclassifications as stomach adenocarcinoma (STAD) occurred in two instances.
(E) Impact of CpG Coverage and Sampling on Diagnostic Sensitivity. To determine if Nanopore's low accuracy was a result of reduced sequencing depth, we downsampled the EPIC array data to match the Nanopore coverage (~3.61% of total CpGs). Despite this significant data reduction, 5/8 FFPE samples remained correctly classified. This markedly outperforms the 1/8 accuracy achieved using true Nanopore FF tissue sequencing on the same cases. These findings suggest that the diagnostic discrepancy is driven less by sequencing depth and more by intraoperative sampling factors, such as low tumor purity in FF samples compared to the curated FFPE blocks used for EPIC profiling.

Supplementary Figure 6

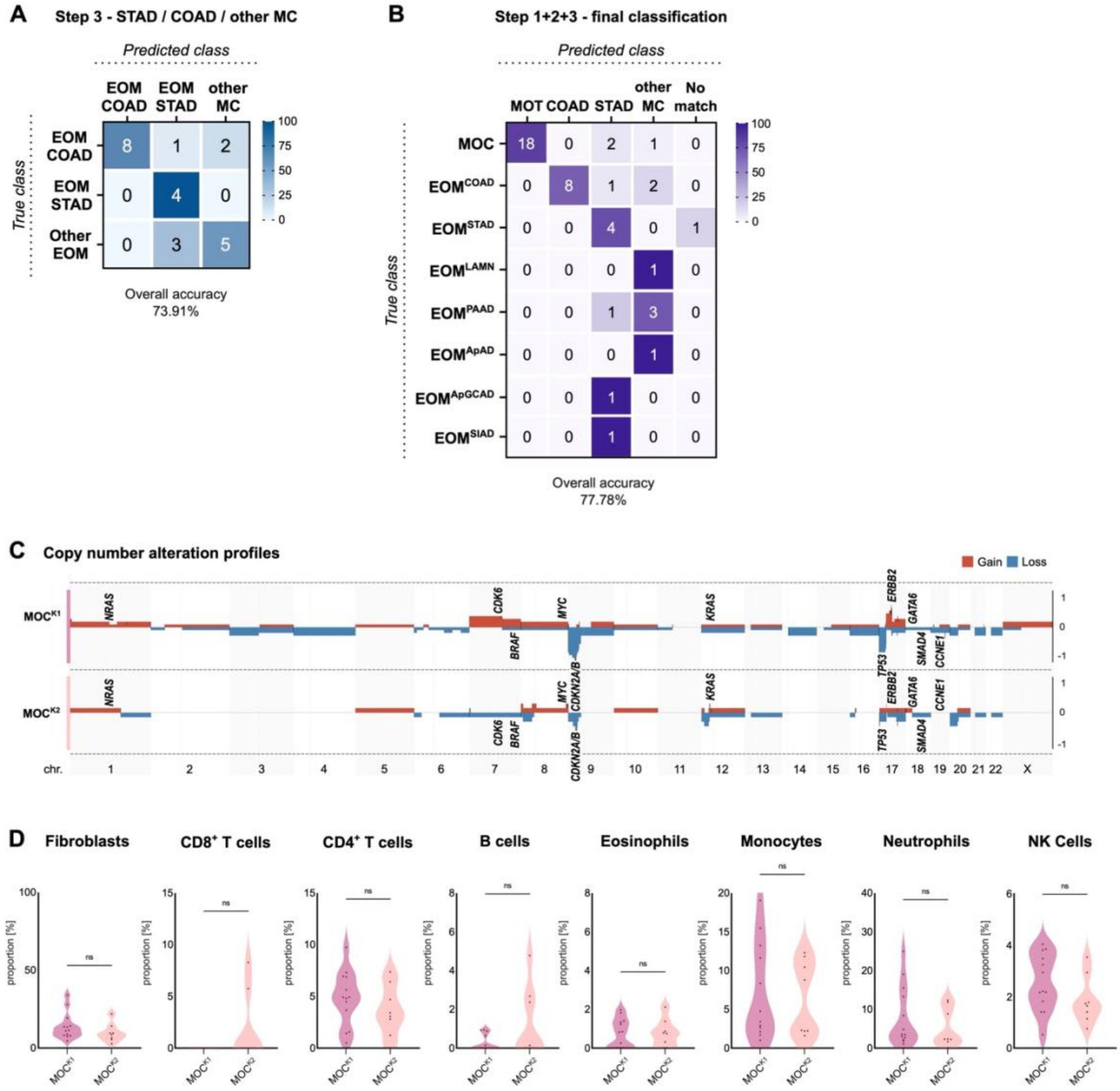

Figure S6. External validation of the classifier and DNA methylation subtypes of MOC. Related to Figures 6.

(A) Confusion matrix showing Step 3 classification results in the external validation cohort.

(B) Confusion matrix summarizing the final three-step DNA methylation classifier (Steps 1-3) in the external validation cohort.

(C) Genome-wide copy number alteration profiles for MOC^{K1} (external validation, n = 13), and MOC^{K2} (external validation, n = 8). The frequency of chromosomal gains (red) and losses (blue) is shown across the genome.

(D) Inferred fractions (proportion of positive cells, %) of fibroblasts, $CD8^+$ T cells, $CD4^+$ T cells, B cells, eosinophils, monocytes, neutrophils, and NK cells across MOC^{K1} (external validation), and MOC^{K2} (external validation), with normal ovary as reference. Statistical comparisons were made using unpaired two-tailed t-test or Mann-Whitney U test, as appropriate. ns, not significant.
