# Supplementary material for: DNA methylation profiling enables subclassification of mucinous ovarian carcinoma and distinguishes it from extraovarian mucinous metastases
Source: Cell Rep Med. 2026 Jul 27;7(8):102941. doi: 10.1016/j.xcrm.2026.102941 (PMC13522789; doi:10.1016/j.xcrm.2026.102941)
Supplement: Supplementary file 2 [file mmc9.pdf]

Cell Reports
Medicine

Article

DNA methylation profiling enables subclassification of mucinous ovarian carcinoma and distinguishes it from extraovarian mucinous metastases

Graphical abstract

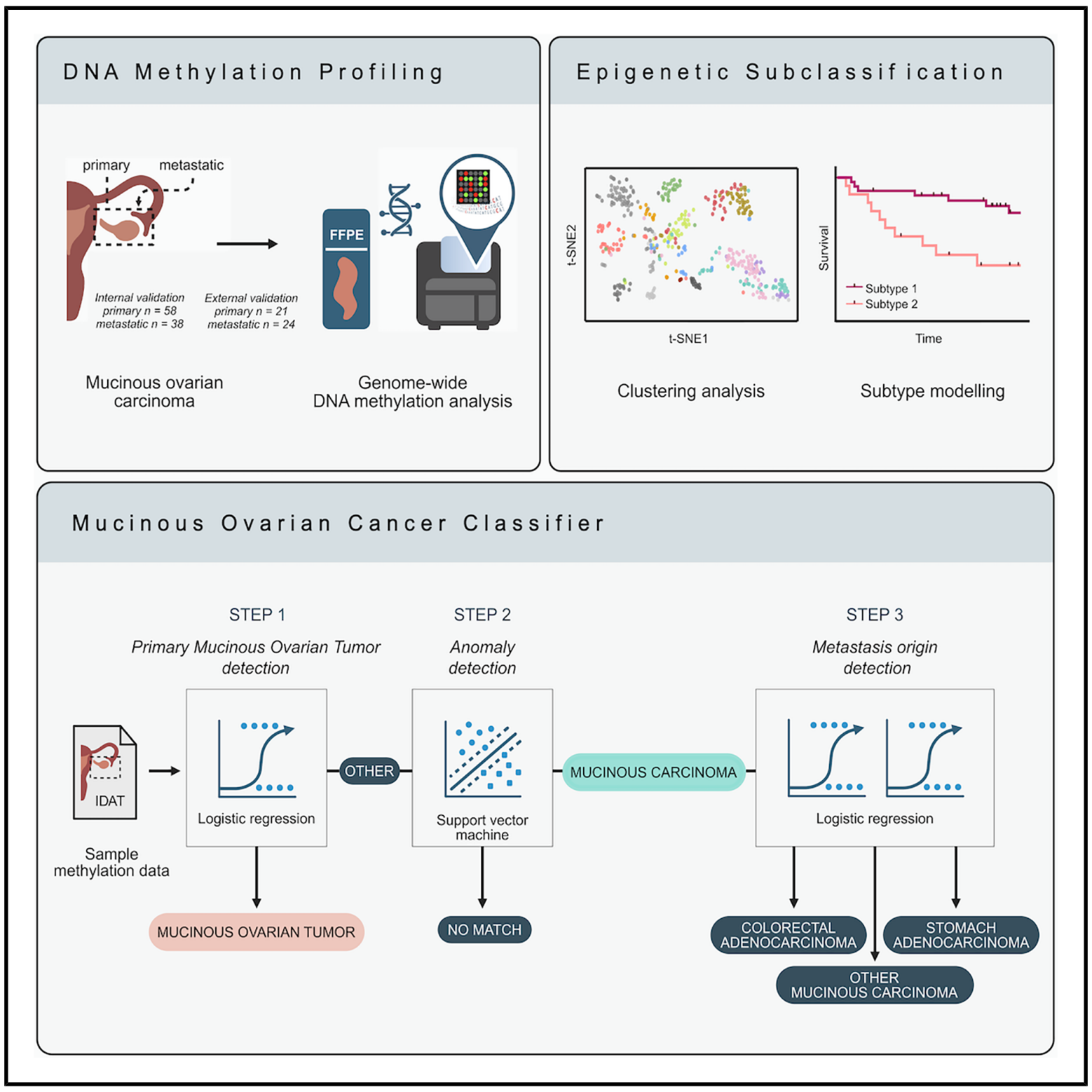

Highlights

- DNA methylation profiling of mucinous ovarian tumors (MOCs/mBOTs) and metastases
- mBOTs separate into “normal-like” and “carcinoma-like” methylation states
- Two epigenetic subtypes of MOC suggest differential survival associations
- Epigenetic classifier achieves >90% accuracy in separating MOCs from metastases

Authors

Teodor G. Calina, Elena Grafenhorst, Simon Schallenberg, ..., Kylie L. Gorringe, David Capper, Mihnea P. Dragomir

Correspondence

mihnea.dragomir@charite.de

In brief

Calina and Grafenhorst et al. using DNA methylation profiling of mucinous ovarian tumors characterize two mucinous ovarian carcinoma (MOC) subgroups with potential differing outcomes and describe epigenetic patterns consistent with the stepwise relationship between mucinous borderline ovarian tumor and MOC. An epigenetic classifier accurately distinguishes MOC from metastases, improving diagnosis.

Calina et al., 2026, Cell Reports Medicine 7, 102941
August 18, 2026 © 2026 The Authors. Published by Elsevier Inc.
https://doi.org/10.1016/j.xcrm.2026.102941

CellPress

Cell Reports Medicine

CellPress
OPEN ACCESS

Article

DNA methylation profiling enables subclassification of mucinous ovarian carcinoma and distinguishes it from extraovarian mucinous metastases

Teodor G. Calina,[1,2,24] Elena Grafenhorst,[3,24] Simon Schallenberg,[3] Markus Möbs,[3] Bjarne Daenekas,[4,5] Alexandru A. Sabo,[6] BaoQing Chen,[7,8] Ines Koch,[3] Tobias Janik,[3] Michael Churchman,[9] Charlie Gourley,[9] Elizabeth L. Christie,[10,11] Catalin Vasilescu,[12] Vlad Herlea,[13] Wolfgang D. Schmitt,[3] Nicolae Leopold,[1] Florian Heitz,[14] Angelina Vlaski,[15] Yuri Tolkach,[16] Felix Bremmer,[17] Frederick Klauschen,[3,4,15] Reinhard Büttner,[16] Philipp Ströbel,[17] Eike Burandt,[18] Guido Sauter,[18] Frank L. Heppner,[5,19] Eliane T. Taube,[3,23] Jalid Sehouli,[20,21] Ioana E. Braicu,[20,21] Philipp Euskirchen,[4,5] David Horst,[3,4] Kylie L. Gorringe,[10,11] David Capper,[4,5] and Mihnea P. Dragomir[3,4,22,25,*]

[1]Faculty of Physics, Babeş-Bolyai University, Cluj-Napoca, Romania
[2]TGC Ventures UG, Berlin, Germany
[3]Institute of Pathology, Charité-Universitätsmedizin Berlin, Corporate Member of Freie Universität Berlin, Humboldt-Universität zu Berlin, Berlin, Germany
[4]German Cancer Consortium (DKTK), Partner Site Berlin, and German Cancer Research Center (DKFZ), Heidelberg, Germany
[5]Department of Neuropathology, Charité-Universitätsmedizin Berlin, Corporate Member of Freie Universität Berlin, Humboldt-Universität zu Berlin, Berlin, Germany
[6]Department of Pediatric Cardiology, Pulmonology and Pediatric Intensive Care Medicine, University Children's Hospital Tübingen, Tübingen, Germany
[7]State Key Laboratory of Oncology in South China, Collaborative Innovation Centre for Cancer Medicine, Guangdong Esophageal Cancer Institute, Guangzhou, China
[8]Department of Radiation Oncology, Sun Yat-sen University Cancer Center, Guangzhou, China
[9]Nicola Murray Centre for Ovarian Cancer Research, CRUK Scotland Centre, Institute of Genetics and Cancer, University of Edinburgh, Edinburgh, Scotland, UK
[10]The Sir Peter MacCallum Department of Oncology, University of Melbourne, Melbourne, VIC, Australia
[11]Peter MacCallum Cancer Centre, Melbourne, VIC, Australia
[12]Department of Surgery, Fundeni Clinical Institute, Bucharest, Romania
[13]Department of Pathology, Fundeni Clinical Institute, Bucharest, Romania
[14]Department of Gynecology and Gynecologic Oncology, Evangelische Kliniken Essen-Mitte, Essen, Germany
[15]Institute of Pathology, Ludwig-Maximilians-Universität Munich, Munich, Germany
[16]Institute of Pathology, University Hospital Cologne, Köln, Germany
[17]Institute of Pathology, University Medical Center Göttingen, Göttingen, Germany
[18]Institute of Pathology, University Medical Center Hamburg-Eppendorf, Hamburg, Germany
[19]German Center for Neurodegenerative Diseases (DZNE), Berlin, Germany
[20]Department of Gynaecology, European Competence Center for Ovarian Cancer, Charité-Universitätsmedizin Berlin, Corporate Member of Freie Universität Berlin, Humboldt-Universität zu Berlin, Berlin, Germany
[21]North Eastern German Society for Gynecological Cancer, Tumor Bank Ovarian Cancer Network, Berlin, Germany
[22]Berlin Institute of Health, Berlin, Germany
[23]Present address: Institute of Pathology, University Hospital Magdeburg, Magdeburg, Germany
[24]These authors contributed equally
[25]Lead contact
*Correspondence: mihnea.dragomir@charite.de
https://doi.org/10.1016/j.xcrm.2026.102941

SUMMARY

Mucinous ovarian carcinoma (MOC) is an epithelial ovarian cancer subtype that is frequently misclassified as extraovarian mucinous metastasis (EOM) because of overlapping features. To address this diagnostic challenge, we perform genome-wide DNA methylation profiling of 58 MOCs, 38 EOMs, and 18 mucinous borderline ovarian tumors (mBOTs) collected from six institutions. Methylation analysis defines two mBOT groups, one epigenetically similar to normal ovary and one resembling MOC. Unsupervised clustering reveals two distinct MOC methylation subtypes with potential prognostic relevance in the internal cohort. Using these data together with 389 external profiles, we develop and validate a three-step machine-learning classifier that distinguishes MOC from EOM with 95.5% accuracy. External validation of this classifier on 21 MOCs and 24 EOMs yields an accuracy of 91.11% for differentiating MOC from EOM. These findings establish an epigenetic framework for mucinous ovarian tumors and provide a robust clinical classification tool.

© 2026 The Authors. Published by Elsevier Inc.
This is an open access article under the CC BY license (http://creativecommons.org/licenses/by/4.0/).

INTRODUCTION

Mucinous ovarian carcinoma (MOC) is one of the rarest and least studied epithelial ovarian cancers. Two aspects make its surgical pathology particularly compelling: the unknown origin and the clinical challenge of distinguishing primary MOCs from metastatic mucinous tumors involving the ovary.

The cell of origin remains uncertain. The prevailing hypothesis suggests that MOC arises from mucinous borderline ovarian tumors (mBOTs), which themselves develop from precursor benign mucinous lesions such as mucinous cystadenomas or adenofibromas.[1] A key argument supporting this theory is the frequent presence of *KRAS* mutations across benign, borderline, and malignant mucinous lesions, indicating a molecular continuum of progression as well as the presence of all three morphological types within a tumor.[1] However, despite this proposed pathogenesis, the true origin of mucinous ovarian lesions remains elusive[2,3] and has not been explored from an epigenetic perspective. Given that the normal ovarian surface epithelium lacks mucin-secreting cells, several origins have been proposed. These include mucinous metaplasia of the ovarian surface epithelium,[4] a germ cell origin—supported by associations between MOC and teratomas[3,5]—and Brenner tumors, which frequently contain mucinous epithelium.[2,3,6] To support our approach of epigenetic characterization of MOC, we recently showed that mucinous cystic neoplasms (MCNs) of the liver and pancreas share an epigenetic profile with MOC,[7] suggesting a possible common origin. This finding highlights the potential of DNA methylation to offer additional insights into the pathogenesis of MOC. In addition, we consider that an epigenetic characterization of MOC could also help the subclassification of these tumors.

The ovary is a common site for extraovarian metastases (EOMs) of mucinous tumors, which account for approximately 10%–25% of malignant ovarian neoplasms.[8,9] These metastases originate from various primary sites outside the ovary.[8–10] Distinguishing primary MOC from EOM remains a significant diagnostic challenge. Retrospective studies have shown that, upon rigorous histopathological reassessment, 50%–70% of tumors initially diagnosed as MOC are reclassified as EOM.[11] Current diagnostic approaches rely on clinical features, histopathology, and immunohistochemistry (IHC) but are limited in accuracy. Clinically, EOMs tend to be bilateral, smaller in size, and more infiltrative, whereas MOCs are typically unilateral, larger, and form solid and cystic masses. Histologically, EOMs often show stromal desmoplasia, infiltrative and nodular growth, ovarian surface involvement, and lymphovascular invasion.[12,13] In contrast, MOCs display a more glandular architecture, often resembling mucinous cystadenomas or mBOTs.[12,13] IHC analysis includes several markers that provide supportive evidence in differentiating MOCs from EOMs. However, a definitive diagnosis based solely on these parameters remains difficult, particularly for upper gastrointestinal primaries.[14] Therefore, there is a pressing need for diagnostic tools that can distinguish MOCs from EOMs. Furthermore, in many cases, EOMs are often detected without an identifiable primary tumor, prompting a complex and time-consuming diagnostic workup to determine their origin and delaying treatment initiation. Thus, a tool capable of predicting the site of the primary tumor would not only streamline the diagnostic process but also accelerate treatment initiation.

DNA methylation patterns are tissue-specific, making them an ideal tool for characterizing rare entities such as MOC and for distinguishing MOCs from EOMs. In recent years, DNA methylation profiling has revolutionized tumor classification, most notably in neuro-oncology, where it has improved the diagnosis of brain tumors.[15] Moreover, methylation-based approaches have facilitated the development of algorithms capable of identifying the tissue of origin in cancers of unknown primary[16,17] and have enabled machine-learning-based diagnostic tools that differentiate primary tumors from metastatic lesions with high accuracy.[18,19] These advances highlight the potential of DNA methylation profiling to resolve the long-standing challenge of differentiating MOCs from EOMs.

RESULTS

The DNA methylation profile of MOC in the context of ovarian tumors

To obtain an epigenetic perspective on MOC, we established a multi-institutional cohort of 58 MOCs and performed a broad IHC and mutational analysis in parallel with genome-wide DNA methylation profiling (Figure 1A). Samples were collected from Charité-Universitätsmedizin Berlin, Germany (Charité; n = 34), Evangelische Kliniken Essen-Mitte, Germany (EKEM; n = 6), and the Peter MacCallum Cancer Center collective, Melbourne, Australia (PMCC; n = 18). The PMCC collective comprised samples from the Australian Ovarian Cancer Study (AOCS; n = 4), the Edinburgh Cancer Research Centre (ECRC; n = 12), the Mayo Clinic (Mayo; n = 1), and the Victorian Cancer Biobank (VCB; n = 1). All samples but one were from formalin-fixed, paraffin-embedded (FFPE) material, while the remaining sample was from fresh-frozen (FF) tissue. The mean patient age at diagnosis was 52 years, while the mean sample age was 12.1 years (Figure 1B).

For all samples from Charité and EKEM, we performed a 12-marker IHC analysis to reevaluate and characterize the cases. All diagnoses were confirmed by the study pathologist based on histology, IHC, clinical data, and sequencing data. Regarding the PMCC samples, all diagnoses were confirmed based on similar criteria.[1] Hence, all 58 samples represent *bona fide* MOCs. Twenty samples were G1 (well-differentiated), 27 G2 (moderately differentiated), and the remaining 11 were G3 (poorly differentiated) (Figures 1B and 1C). The morphological growth patterns were classified as either infiltrative or expansile subtypes, based on established criteria,[20,21] resulting in 42 expansile and 11 infiltrative cases (Figure 1B). We stratified cases using IHC profiles following published algorithms.[22–25] In total, 30 samples displayed an MOC-indicative profile and 6 were MOC-unspecific (Figure 1B; Figure S1A).

Next, we analyzed a cohort of 18 mBOT Charité cases. All were FFPE, with a median sample age of 1.6 years and a median patient age of 54.1 years. All but one were stage T1, the other was T2. All diagnoses were confirmed by the study pathologist after assessment of clinical, histological, IHC, and mutational data (Figure 1B).

CellPress
OPEN ACCESS

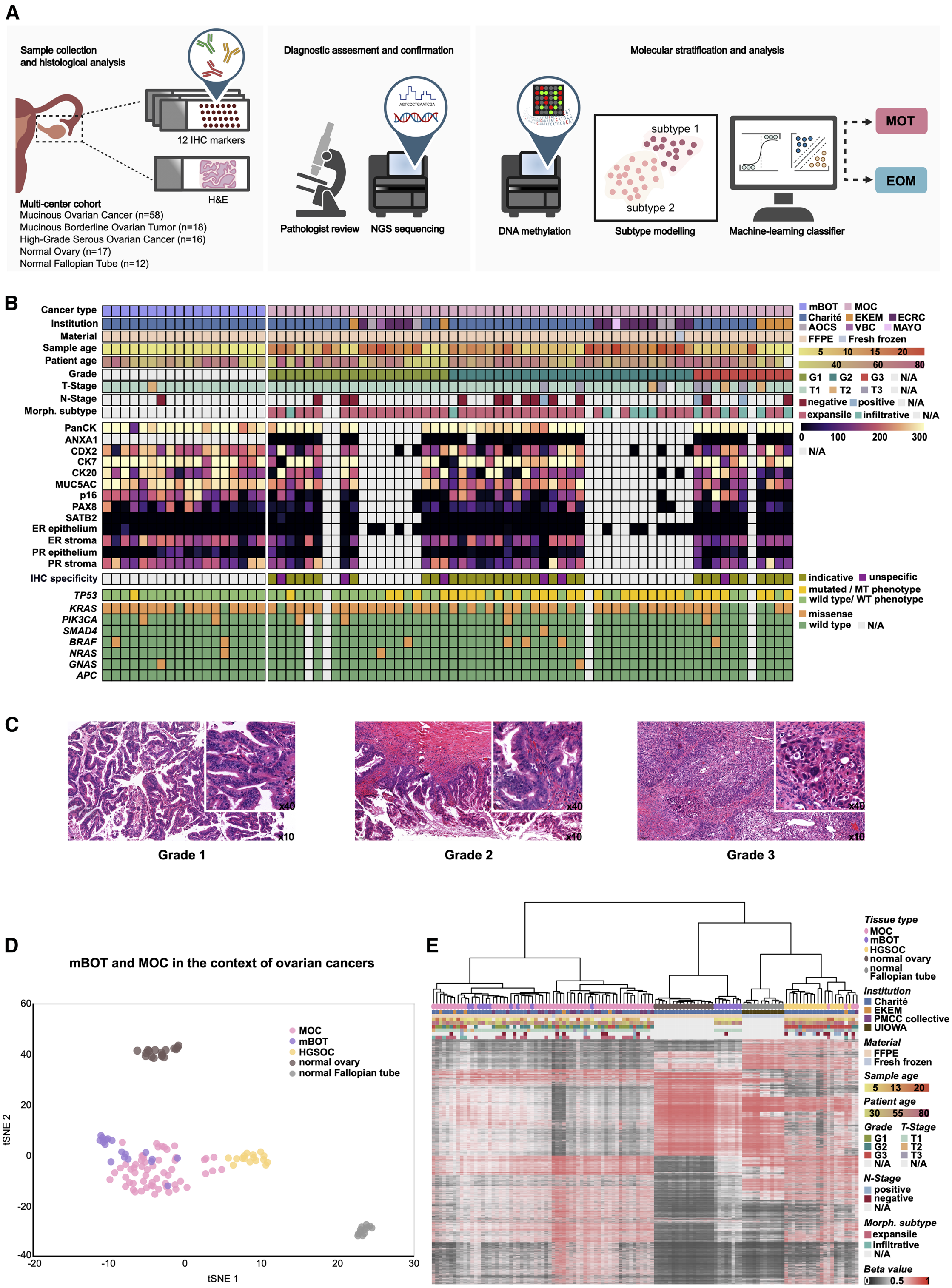

Figure 1. The DNA methylation profile of MOC in the context of ovarian tumors

(A) Workflow for DNA-methylation-based characterization and development of a machine-learning classifier to distinguish mucinous ovarian tumors (MOTs) from EOMs.

Cohort characterization included 12-marker IHC and targeted DNA sequencing. Created with BioRender.com.

(B) Clinicopathological, immunohistochemical, and mutational features of mBOT and MOC.

(legend continued on next page)

To minimize heterogeneity, we extracted DNA exclusively from ovarian tumor blocks in all cases. For 37/40 samples in the Charité/EKEM subcohort, we performed targeted DNA sequencing, while for the 17/18 PMCC cases, mutations were retrieved from the prior publication.[1] Because the targeted sequencing panel only partially covered *TP53*, we additionally employed IHC for p53 to infer the samples with a mutant phenotype. As expected,[1,26–28] *KRAS* and *TP53* were the most commonly mutated genes, with an enrichment of *TP53* mutations in G2 and G3 samples (Fisher exact test, $p = 0.0021$) and an almost equal distribution of *KRAS* mutations across grades (Fisher exact test, $p = 0.1203$). Furthermore, four samples harbored *PIK3CA* mutations, one sample *SMAD4* mutation, five *BRAF* mutations, and one sample *GNAS* mutation (Table S1). All these mutations were previously reported for MOC.[26–28] *KRAS* was the most frequently mutated gene in mBOT, present in 16/18 samples. *PIK3CA* and *BRAF* were mutated in 2/18 each, while *NRAS* and *GNAS* appeared in 1/18 each. *TP53* was mutated in 1/18 mBOTs.

For all mBOT and MOC samples, we performed genome-wide DNA methylation profiling. Using the MOC cohort, the 18 mBOTs, 16 high-grade serous ovarian carcinoma (HGSOCs), 17 normal ovaries, and 12 normal Fallopian tubes, we created an epigenetic landscape of ovarian tumors. MOCs formed a distinct and mostly homogeneous subgroup, separate from HGSOC (Figure 1D). Furthermore, normal Fallopian tube, normal ovary, and approximately half of the mBOT samples formed three distinct groups, while the remaining mBOT samples grouped with MOC.

Next, we examined parameters that could impact sample distribution and noted that institution, sample age, grade, T-stage, IHC-specificity profile, *KRAS*, *TP53*, *BRAF* mutation status, and precursor lesions had no impact on sample grouping (Figures S1B–S1J). Tumor purity, however, showed a gradient across MOCs in the t-SNE, and, for the mBOT samples, we clearly observed differences in tumor purity between the distinct group and the samples grouping with MOCs (Figure S1K). To validate purity estimates, we compared our results with values inferred by doubling the allele frequency of oncogenic mutations, most often *KRAS* (or *BRAF*/*NRAS* when *KRAS* was absent), and found a strong concordance (Spearman r test, $p < 0.0001$).

Furthermore, six MOC samples grouped closer to HGSOC (Figure 1D), consistent with prior pan-ovarian DNA methylation analysis.[29] These cases were not enriched in any characteristic except for being slightly older than the general cohort samples (Figures S1B–S1J).

Using a second method for characterizing the samples based on DNA methylation, hierarchical clustering confirmed two mBOT groups: one intermingled with MOC and another forming distinct, near-normal samples. Additionally, MOC samples showed a distinct methylation profile relative to other tumors but separated internally into two distinct clusters, suggesting the existence of two potential epigenetic MOC subtypes (Figure 1E).

DNA methylation defines molecular subtypes of MOC and mBOT

The DNA methylation profiling of samples described above defined several groups: normal ovary, $mBOT^{normal\_like}$, $mBOT^{MOC\_like}$, and MOC. By comparing the two mBOT subgroups, we did not observe any significant differences in patient age or mutational parameters. However, $mBOT^{normal\_like}$ showed significantly higher progesterone receptor (PR) and p16 expression versus $mBOT^{MOC\_like}$, whereas all other IHC parameters showed no difference between the two subtypes (Table S2). Morphological reanalysis revealed a more complex morphology and cytology for $mBOT^{MOC\_like}$ compared to $mBOT^{normal\_like}$ (Figure S2A). This observation confirms our previous analysis of MCNs in the context of ovarian cancers, in which some mBOTs grouped with low-grade MCN of the pancreas/liver, while others aligned with high-grade and invasive MCNs.[7]

Furthermore, we performed unsupervised K-means clustering of MOC samples, identifying two clusters: MOC^{K1} and MOC^{K2} (Figure 2A; Figure S2B). Mapping these clusters onto the landscape t-SNE revealed specific spatial relations (Figure S2C) that were not easily discernible previously (Figure 1D). Notably, we observed that most (9/10) $mBOT^{MOC\_like}$ grouped with MOC^{K1}, forming a continuous progression.

We compared MOC^{K1} and MOC^{K2} by patient age, histology, IHC marker expression and score, mutational profile, and precursor lesions, observing no significant statistical difference (Table S3). However, consistent with the tumor purity distribution, a significant difference between MOC^{K1} and MOC^{K2} was observed (unpaired *t* test, $p < 0.0001$) (Figure S1J; Table S3). Next, as there are very few prognostic parameters for MOC,[30] and in order to check the potential clinical relevance of MOC^{K1} and MOC^{K2}, we assessed whether these epigenetic subtypes were associated with differences in overall survival (OS). The median cohort OS was 9.1 years. Patients with MOC^{K1} exhibited longer OS compared to those in MOC^{K2} (log rank test, $p = 0.0059$; Figure 2B). Patient age, all available pathological, IHC, and molecular parameters had no impact on the OS, except for T-stage, which showed a notable significance (log rank test, $p = 0.0006$; Figure S2D; Table S4). In a multivariate Cox proportional hazards analysis, both advanced tumor stage and DNA methylation cluster K2 were independently associated with reduced survival. T2+ tumors were associated with a 4.38-fold increased risk of death, and MOC^{K2} patients had a 3.22-fold increased risk (hazard ratio [HR] 3.22, 95% confidence interval [CI] 1.23–8.42, $p = 0.017$), independent of tumor stage (Figure 2C).

Therefore, we focused on understanding the epigenetic characteristics of the newly observed mBOT and MOC subgroups and their epigenetic relationship to normal ovary. We

(C) Representative hematoxylin and eosin (H&E) images of MOC G1, G2, and G3 (scale bars, 100 μm; ×10 and ×40 magnification).
(D) t-SNE representation of DNA methylation profiles for mBOT, MOC, HGSOC, normal ovary, and normal Fallopian tube, based on the top 20,000 most variable CpGs.
(E) Hierarchical clustering and heatmap of DNA methylation profiles for the same tumor and normal samples as in (D), based on the top 20,000 most variable CpGs.

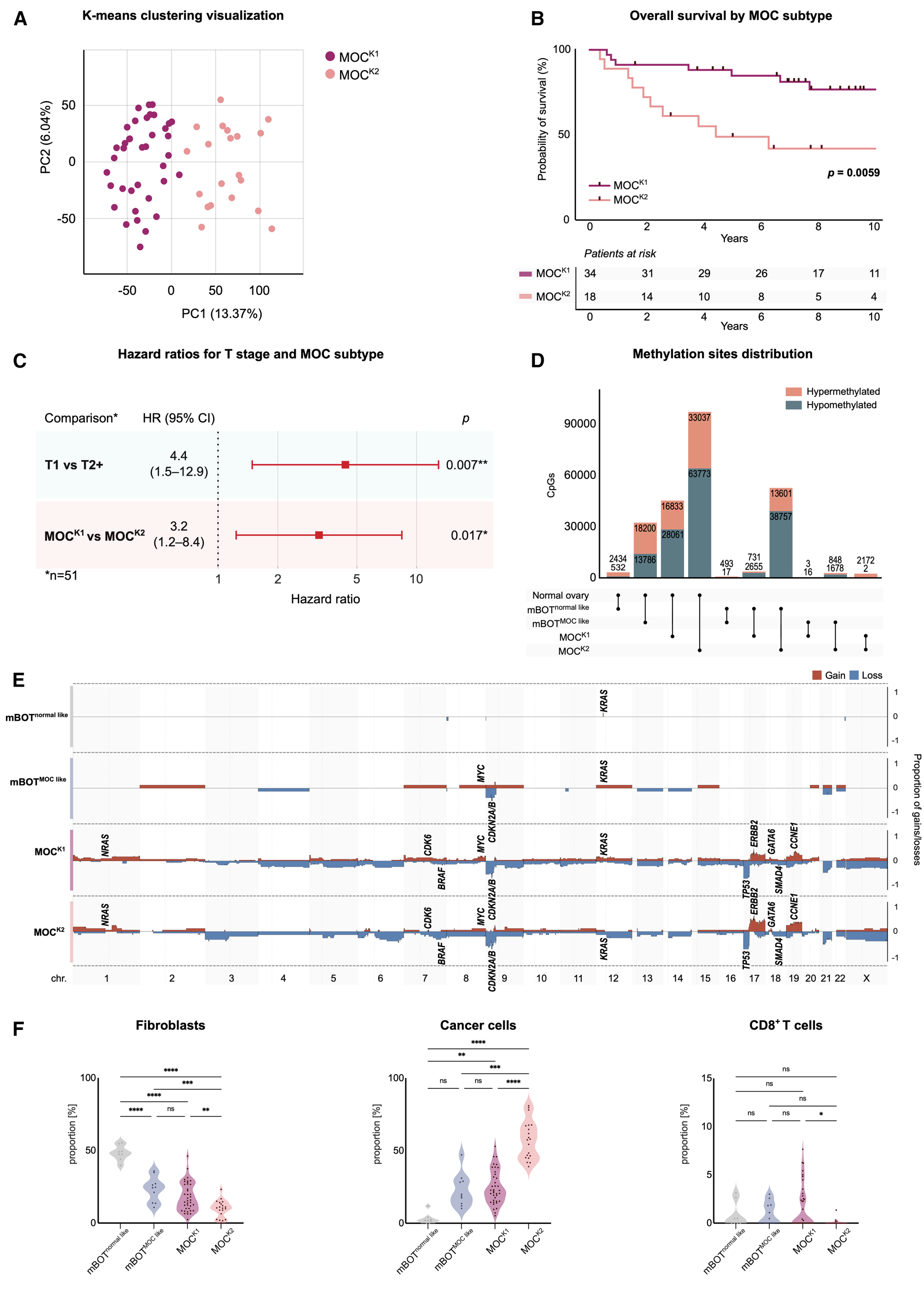

Figure 2. DNA methylation defines molecular subtypes of MOC and mBOT

(A) Principal-component analysis (PCA) of MOC samples colored by K-means clustering, separating MOC^{K1} and MOC^{K2} subtypes. The percentage of variance explained by PC1 and PC2 is indicated on the axes.

(B) Kaplan-Meier OS curves for patients with MOC^{K1} versus MOC^{K2} (n = 52). p value from log rank test.

(legend continued on next page)

hypothesized that $mBOT^{MOC\_like}$ have already acquired most of the molecular characteristics of an MOC.

First, we performed differentially methylated probe (DMP) analysis pairwise among the four groups and normal ovary. We observed a clear *crescendo* in the absolute number of DMPs, progressing from $mBOT^{normal\_like}$ versus normal ovary (2,966 DMPs), to $mBOT^{MOC\_like}$ versus normal (31,986 DMPs), to MOC^{K1} versus normal (44,894 DMPs), and finally to MOC^{K2} versus normal (96,810 DMPs) (Figure 2D; Figure S2F). This analysis further revealed minimal differences between $mBOT^{MOC\_like}$ and MOC^{K1} (only 19 DMPs) (Figure 2D). Using DMPs, we built an epigenetic relatedness network, where each edge represents the number of DMPs between two groups. While the t-SNE and clustering analysis were performed using the most standard deviated CpGs, this analysis is based on a completely different CpG prioritization, further showing the closest epigenetic relationship between $mBOT^{MOC\_like}$ and MOC^{K1}, as well as close relationships between the two types of mBOTs, including $mBOT^{normal\_like}$ with MOC^{K1}, $mBOT^{MOC\_like}$ with MOC^{K2}, MOC^{K1} with MOC^{K2}, and normal ovary with $mBOT^{normal\_like}$ (Figure S2E). By contrast, HGSOC and normal Fallopian tube were more distant from mucinous ovarian tumors (MOTs) (Figure S2E).

Second, to further investigate the relationship between dysregulated methylation and tumor progression, we analyzed the differentially methylated pathways across the groups (Table S5). Pathways related to apoptosis and cell death were activated in $mBOT^{normal\_like}$ compared to normal ovary. Comparing $mBOT^{normal\_like}$ to $mBOT^{MOC\_like}$, we identified a few differentially methylated pathways, all of which were inactivated, including O-linked glycosylation of mucins. Aberrant glycosylation has previously been hypothesized to play a role in cancer progression.[31] Interestingly, the epigenetic differences between $mBOT^{MOC\_like}$ and MOC^{K1} were minimal. In contrast, a larger number of dysregulated pathways distinguished $mBOT^{MOC\_like}$ and MOC^{K2}, including activation of O-linked glycosylation and inhibition of G-protein-coupled receptor (GPCR)-related signaling (Table S5) in MOC^{K2}. GPCR dysregulation has been previously reported in MOC and was associated with poorer survival outcomes.[32]

Third, we performed summary copy number analysis (CNA) plots for the two subtypes of mBOT and MOC. $mBOT^{normal\_like}$ presented an almost flat copy-number profile, with only one sample showing a clear *KRAS* gain. $mBOT^{MOC\_like}$ showed *CDKN2A/B* loss in 4/10 samples, which could also be observed in most MOC^{K1} and MOC^{K2}. In MOC^{K1}, we noticed a gain of chromosome 17q that included the *ERBB2* gene (16/38 samples, 42.11%) and a loss of 17p that included *TP53* (22/38 samples, 57.89%). These chromosomal changes were also present in MOC^{K2}, *ERBB2* gain (8/20, 40%), and *TP53* loss (11/20, 55%) and were previously reported especially in high-grade MOCs.[1,33] Finally, we noticed a large gain of most of chromosome 19, which was more pronounced in MOC^{K2} compared to the other groups (Figure 2E). This region includes the *CCNE1* gene that was recently associated with poor prognosis in multiple epithelial ovarian cancers[34] and was gained in 10/38 (26.32%) MOC^{K1} and 6/20 (30%) MOC^{K2} cases.

Finally, we deconvoluted the cellular composition using MethylCIBERSORT.[35,36] We observed a gradual decrease in fibroblasts and a gradual increase in cancer cells from $mBOT^{normal\_like}$ toward MOC^{K2} (Figure 2F). Specifically, fibroblast proportions were highest in $mBOT^{normal\_like}$ and decreased noticeably in both MOC^{K1} (one-way ANOVA test, $p < 0.0001$) and MOC^{K2} (one-way ANOVA test, $p < 0.0001$). Conversely, cancer cell proportions were significantly higher in MOC^{K1} (Kruskal-Wallis test, $p = 0.0056$) and MOC^{K2} (Kruskal-Wallis test, $p < 0.0001$) compared to $mBOT^{normal\_like}$ samples. In addition, we observed significantly higher levels of $CD8^{+}$ T cells in MOC^{K1} versus MOC^{K2} (Kruskal-Wallis test, $p = 0.0414$) (Figure 2F). Spatial IHC analysis confirmed these $CD8^{+}$ differences: MOC^{K1} tumors exhibited more intra-tumoral infiltration, whereas in MOC^{K2}, $CD8^{+}$ positivity was more dominant in the stromal compartment (Mann-Whitney test, $p = 0.0452$) (Figure S2H). This suggests that for MOCs, an immune-enriched subtype (MOC^{K1}) shows better survival rates, similar to HGSOC.[37] For the other immune cells, we found no significant differences (Figure S2G).

DNA methylation profiling of EOM in the context of ovarian tumors

To develop a machine-learning algorithm that differentiates MOC from EOM, we assembled a cohort of mucinous metastases to the ovary. We collected 38 EOM samples (Figure 3A), including appendiceal adenocarcinomas (ApAD, $n = 3$), low-grade appendiceal mucinous neoplasm (LAMN, $n = 3$), cholangiocarcinoma (CHOL, $n = 2$), colorectal adenocarcinoma (COAD, $n = 9$), esophageal adenocarcinoma (EAC, $n = 1$), lung adenocarcinoma (LUAD, $n = 2$), and stomach adenocarcinoma (STAD, $n = 18$) (Figure 3B). All samples were FFPE, and all but one were from Charité. The mean age of the patients at diagnosis was 53.7 years, while the mean sample age was 7.8 years. For each case, we performed IHC analysis (35 showed IHC profiles consistent with EOM and 3 were unspecific) and corroborated the results with the clinical history, thus we have carefully confirmed the EOM diagnosis.

Importantly, all analyses were performed on tissue from the ovarian metastasis site. Targeted DNA sequencing and IHC

(C) Forest plot summarizing multivariate Cox proportional hazards analysis for OS.
Dots indicate hazard ratios, and horizontal lines show 95% confidence intervals; p values from Wald tests.
(D) Distribution of DMPs identified in pairwise comparisons among normal ovary, $mBOT^{normal\_like}$, $mBOT^{MOC\_like}$, MOC^{K1}, and MOC^{K2}.
Bars show numbers of hypermethylated (orange) and hypomethylated (green) CpGs.
(E) Genome-wide copy-number alteration profiles for $mBOT^{normal\_like}$ ($n = 8$), $mBOT^{MOC\_like}$ ($n = 10$), MOC^{K1} ($n = 38$), and MOC^{K2} ($n = 20$).
The frequency of chromosomal gains (red) and losses (blue) is shown across the genome.
(F) Inferred fractions (%) of fibroblasts, cancer cells, and $CD8^{+}$ T cells across $mBOT^{normal\_like}$, $mBOT^{MOC\_like}$, MOC^{K1}, and MOC^{K2}, with normal ovary as reference.
Each dot represents a unique patient.
Statistical comparisons were made using Kruskal-Wallis or one-way ANOVA, as appropriate. ns, not significant; $^{*}p < 0.05$; $^{**}p < 0.01$; $^{***}p < 0.001$; $^{****}p < 0.0001$.

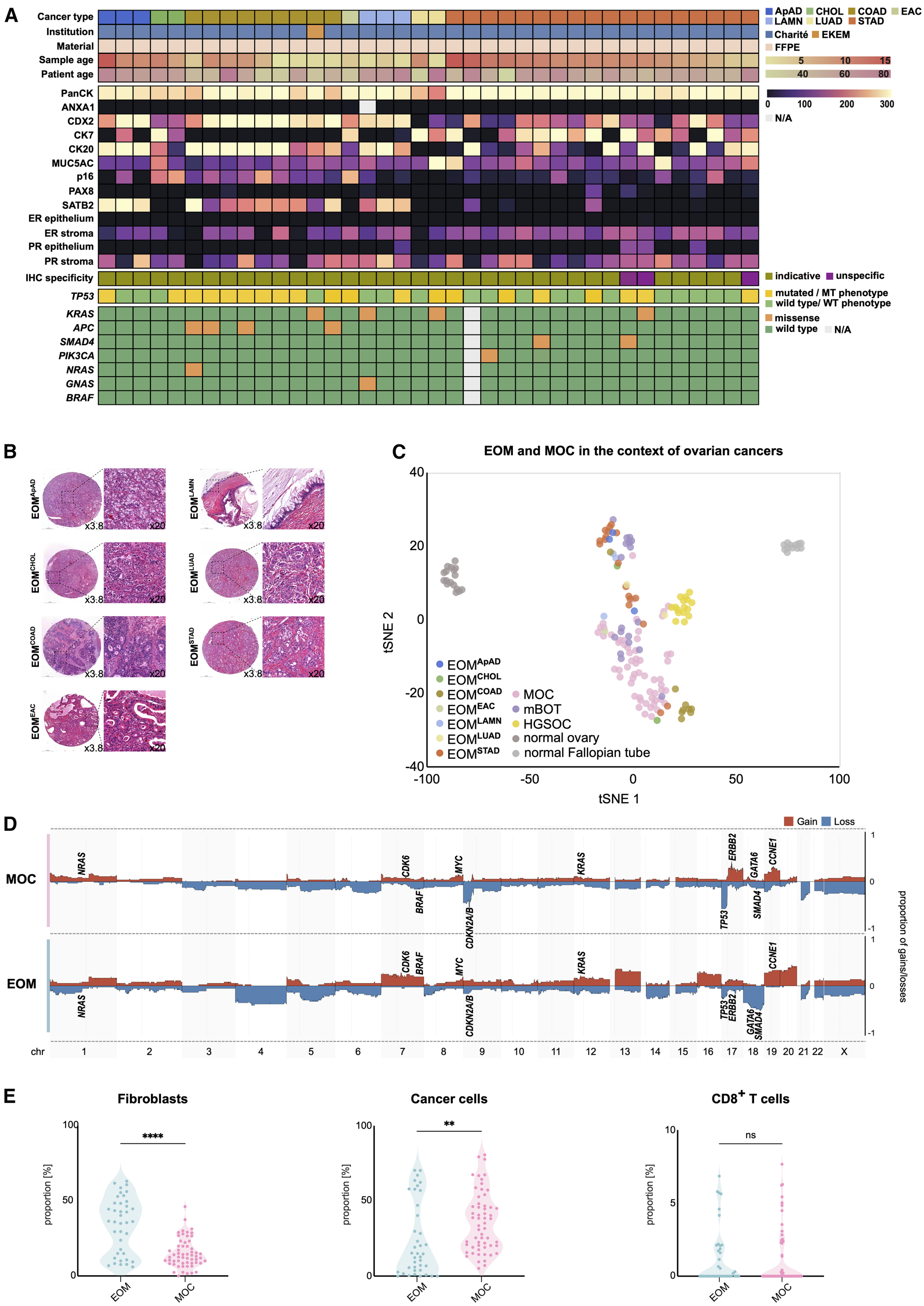

(legend on next page)

revealed *TP53* mutations or mutant p53 phenotype in 20/37 (54.05%) cases. Other recurrent mutations included *KRAS* (n = 4), *APC* (n = 4), *SMAD4* (n = 2), and single cases with *PIK3CA*, *NRAS*, or *GNAS* (Figure 3A).

When comparing the different parameters between MOCs and EOMs, we found a significantly higher histologic grade and increased CDX2 expression in EOMs, while MOCs more frequently expressed CK7, MUC5AC, PAX8, and PR. At the mutational level, *KRAS* mutations were significantly more common in MOC, whereas *TP53* frequency was similar (Table S6).

We then performed genome-wide DNA methylation profiling for all 38 EOMs and projected them with the existing ovarian tumor t-SNE (Figure 3C). EOMs tended to form spatially distinct groups apart from MOCs, with EOM^{COAD} samples most separated (Figure 3C). Sample distribution was not influenced by patient age, tumor primary site, or mutational spectrum but was partially influenced by tumor purity (Figures S3A–S3F). Because EOM^{STAD} samples frequently exhibit signet ring morphology, this lower purity was somewhat expected (Figure S3F; Figure 3B).

CNA profiling highlighted key differences between MOC and EOM groups. As already reported in the literature, MOC showed more frequent *CDKN2A/B* losses (MOC: 55.17% vs. EOM: 36.84%),[26] and, in addition, we observed an enrichment in losses of the *TP53* region (MOC: 56.9% vs. EOM: 31.58%) and a gain of *ERBB2* (MOC: 41.38% vs. EOM: 10.53%). On the other hand, we observed more frequent gains of entire chromosomes (chrs. 7, 13, 19, and 20) in the EOM group versus MOC group (Figure 3D).

We also compared the cellular composition after DNA methylation deconvolution. We observed a higher proportion of fibroblasts (Mann-Whitney test, $p < 0.0001$) and lower level of cancer cells (Mann-Whitney test, $p = 0.0014$) in EOM versus MOC, and regarding the immune infiltrate we noticed higher levels of neutrophils (Mann-Whitney test, $p = 0.0384$) and lower levels of B cells (Mann-Whitney test, $p = 0.0271$) in MOC versus EOM, while all other immune cells showed no differences (Figure 3E; Figure S3G).

DMP analysis identified 293 significant DMPs between EOM and MOC (Figure S3H). This degree of relatedness resembled that seen between $mBOT^{normal\_like}$ and $mBOT^{MOC\_like}$ (Figure 2D), underscoring the difficulty of distinguishing MOC and EOM. Not surprisingly, genes with hypomethylated CpGs in EOM versus MOC included transcription factors linked to specific tumor types, such as SATB2, a marker of COAD.

A DNA methylation classifier for differentiating EOM from MOC

With the assembled MOC and EOM cohorts, we next tackled one of the most difficult clinical challenges of mucinous carcinomas of the ovary—differentiating between MOC and EOM. To build an accurate and safe DNA methylation classifier, we developed a three-step machine-learning pipeline: step 1 to detect primary MOTs (defined as mBOT+MOC), step 2 to identify EOM by separating mucinous carcinomas (MCs) from all other lesions (anomaly detection), and step 3 to predict the tissue of origin of EOM. Samples not classified as MOTs in step 1 proceed to step 2, while those labeled as MCs in step 2 advance to step 3 (Figure 4A).

For step 1, we used 20 MOC samples and 172 other lesions—including primary MCs (pMCs) that can metastasize to the ovary and show mucinous morphology—as the training set. For step 2, we used 133 pMCs (origin of EOM) and 116 non-mucinous lesions. For step 3, the training data contained the same 133 pMCs from step 2, divided into three classes: COAD (n = 20), STAD (n = 20), and other MCs (n = 93). Internal model validation was performed on an independent cohort of 311 samples, including 38 MOCs, 18 mBOTs, and 38 EOMs, none of which were included in the training or parameter tuning phases (Figure 4B). In a t-SNE analysis containing all samples mentioned above, MOCs, mBOTs, and EOMs largely grouped together and separately from the other tumors, including the tumors of origin of the EOMs. The only exception was EOM^{COAD}, which grouped near the primary COAD group (8/9 samples). Overall, this analysis revealed differences between MOC and EOM, while also highlighting similarities that may partly explain the challenges in their diagnosis (Figure S4A).

For step 1, we selected the top 5,000 most variable CpGs of the training set and compared two algorithms: logistic regression (LR) and support vector machine (SVM) (Figure 4A). LR outperformed SVM for MOT detection, with accuracies of 94.44% versus 83.33% for mBOT and 92.11% versus 89.47% for MOC. For EOMs, SVM outperformed LR: 94.74% compared to 89.47% (Figure 4C). Overall, LR achieved a higher accuracy of 97.11% than SVM 96.78% (Figure 4C). We therefore selected the LR classifier for further analysis. Per-class analysis showed misclassification of three MOCs, one mBOT, four EOMs, and one pMC (Figure 4D). Analyzing the factors associated with the classification results revealed lower accuracy for FFPE versus FF samples, EPICv1 versus EPICv2 arrays, and *in-house* samples versus other datasets (*in-house* samples included metastases) (Figure S4B). We also tested if different probability thresholds would improve accuracy, specificity, and sensitivity and observed that the best results are reached without using any threshold, ensuring predictions for all samples (Figures S4C and S4D).

The goal of step 2 was to detect the MC samples, both primary (pMC) and metastatic (EOM), and filter out all other tumor types. This anomaly detection layer serves as a safeguard, ensuring

Figure 3. DNA methylation profiling of EOM in the context of ovarian tumors

(A) Clinicopathological, immunohistochemical, and mutational features of the EOM cohort.

(B) Representative H&E images of EOM^{ApAD}, EOM^{CHOL}, EOM^{COAD}, EOM^{EAC}, EOM^{LAMN}, EOM^{LUAD}, and EOM^{STAD} (low magnification, ×3.8; high magnification, ×20).

(C) t-SNE representation of DNA methylation profiles for EOM, mBOT, MOC, HGSOC, normal ovary, and normal Fallopian tube, based on the 20,000 most variable CpGs.

(D) Genome-wide copy-number alteration profiles of MOC (n = 58) and EOM (n = 38). Frequencies of chromosomal gains (red) and losses (blue) are shown across the genome.

(E) Inferred fractions (%) of fibroblasts, cancer cells, and $CD8^+$ T cells in EOM and MOC, with normal ovary as reference. Each dot represents a unique patient. Statistical comparisons were made using unpaired two-tailed t test or Mann-Whitney U test, as appropriate. ns, not significant; $^{**}p < 0.01$; $^{****}p < 0.0001$.

CellPress
OPEN ACCESS

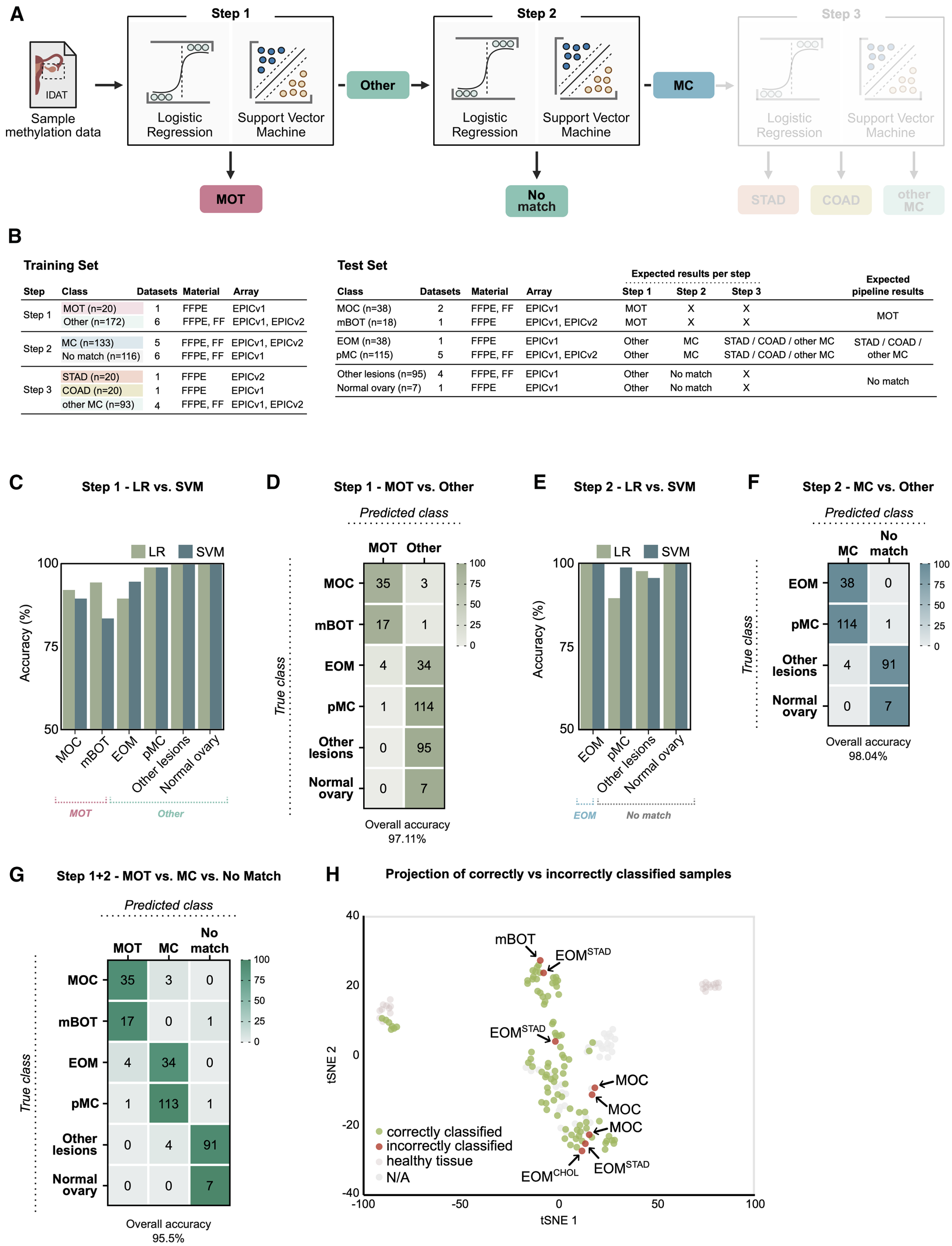

Figure 4. A DNA methylation classifier for differentiating EOM from MOC—step 1 and step 2

(A) Schematic of the three-step classifier workflow.

Step 1 detects MOT. Samples not classified as MOT proceed to step 2, where EOMs are detected and other tumor types are filtered out. Step 3 further resolves the tissue of origin for EOM. Created with BioRender.com.

(B) Overview of training and test cohorts used in each classifier step.

(C) Comparison of step 1 accuracies using LR versus SVM across tumor groups: MOC, mBOT, EOM, primary mucinous carcinomas that can metastasize to the ovary (pMC), other lesions (non-mucinous neoplasms), and normal ovary.

(legend continued on next page)

that only mucinous carcinomas progress to downstream classification. We again developed and compared LR and SVM classifiers. Here, SVM outperformed LR (98.04% vs. 94.51%), especially for pMCs (99.13% vs. 89.57%; Figure 4E). Hence, SVM was chosen as the classification method for step 2. The investigation of misclassified samples showed no EOM cases were incorrectly classified; only one pMC was misclassified, and four non-mucinous lesions were incorrectly labeled as MC (Figure 4F). The SVM performed better for FFPE vs. FF, EPICv2 vs. EPICv1, and for the datasets containing mainly EOMs (*in-house*) and pMC samples (Figure S4E). Again, we observed that the highest accuracy and the best balance between specificity and sensitivity is achieved if no thresholds are applied (Figures S4F and S4G).

These two steps jointly answer the main clinical question: is a mucinous ovarian lesion primary or metastatic? The overall accuracy achieved was 95.5% (Figure 4G), with most misclassified samples being MOCs (3/38) and EOMs (4/38) (Figure S4H). t-SNE mapping showed two misclassified MOC cases grouping near HGSOC, while the rest of the samples were distributed heterogeneously (Figure 4H).

Finally, we compared our data with the results of an IHC classification of the *in-house* samples. Based on IHC, we reached an accuracy of 87.84% (Figure S4I), with only one sample overlapping between IHC and DNA methylation misclassifications, underlining the complementarity of the two tools (Figure S4J).

Exploring the origin of EOM

Next, we analyzed the possibility of expanding our classifier to predict the primary site of EOM. Because sample availability was limited, we trained and tested this step only for the two most frequent origins: gastric (EOM^{STAD}) and colon (EOM^{COAD}), which account for the majority of mucinous metastases to the ovary.[12] For step 3, all samples labeled as MC in step 2 represented the training set for the tissue of origin detection and were divided into three classes: STAD, COAD, and other MC (Figure 5A). Similar to the previous steps, we trained two binomial classifiers to identify STAD and COAD origin, respectively, with other MC classification resulting from the combination of the two models. We again compared LR and SVM models for each classifier and observed higher or equal accuracies for LR when compared independently (STAD: 91.5% [LR] vs. 88.24% [SVM]; COAD: 98.04% [LR] vs. 96.73% [SVM]). We therefore selected LR for both models in step 3. The two classifiers were arranged sequentially, the COAD classifier running first due to the higher accuracy, followed by the STAD classifier. Across the whole testing set, step 3 reaches an accuracy of 92.81%, with an accuracy of 78.94% on just the EOM subset (Figure 5B). Per-sample analysis showed 8/9 EOM^{COAD} correctly labeled as COAD, 11/18 EOM^{STAD} classified as STAD, 11/11 other EOM classified as "other" (Figure 5C).

Next, we analyzed the entire diagnostic pipeline, which yielded an overall accuracy of 92.28% with very few samples misdiagnosed. Of the focal samples, MOTs and EOMs, only three MOCs were misassigned as COAD or STAD, and one mBOT as "no match." Four EOM samples were labeled as MOTs, one EOM^{CHOL} and three EOM^{STAD}. All remaining EOMs were correctly identified as "other" in step 1 and confirmed as MC in step 2 (Figure 5D; Figure S5A).

We also applied the crossNN classifier[38] using rapid Nanopore sequencing of FF tissue for potential intraoperative diagnosis. The Nanopore sequencing showed limited accuracy in classifying MOC FF samples and showed better results on FFPE tissue, even after downsampling EPIC CpGs to match Nanopore coverage (Figures S5B–S5E). Overall, Nanopore-based DNA methylation profiling for intraoperative MOC diagnosis remains challenging, as accurate classification depends critically on selecting tumor-rich regions for DNA extraction.

External validation of the classifier and prognostic tool

To validate our classifier, we analyzed 45 FFPE samples (21 MOCs and 24 EOMs) sourced from three independent institutions (Figure 6A). Hybridization was performed using the Illumina EPICv2 array, a next-generation array not utilized during the classifier development. For step 1 of the classification pipeline, 18 MOC samples were correctly identified as MOT, while 27 were labeled as "other," yielding an initial accuracy of 93.33% (Figure 6B). The step 2 analysis of EOM samples resulted in a classification accuracy of 95.83% (Figure 6C). Critically, the combined step 1 and step 2 pipeline, which addresses the primary clinical diagnostic challenge, achieved an accuracy of 91.11% (Figure 6D). Only four samples were misclassified: three MOC cases, two of which exhibited sarcomatoid morphology with osteoclast-like giant cells, and a single EOM sample, a gastric metastasis, incorrectly labeled as "no match" due to low tumor purity. Step 3 correctly indicated the origin of EOM in 17 out of 23 cases that reached this step, yielding an accuracy of 73.91% (Figure S6A). The lower accuracy was induced by some atypical EOM origins, such as an ileum adenocarcinoma and an appendiceal goblet cell adenocarcinoma, all of which were labeled as STAD. The classifier achieved an overall accuracy of 77.78% in the external validation cohort (Figure S6B), mainly because of step 3. When compared to the MOC and EOM samples of the internal validation cohort, accuracy was similar, although modestly lower than that observed in the full cohort due to the significant number of primary tumors in the internal validation cohort that serve as both positive and negative controls. Step 3 is designed specifically to indicate metastatic origins and narrow the search for the primary tumor and does not represent a pan-adenocarcinoma classifier.

Lastly, we extended our analysis of K1 and K2 MOC clustering to the external cohort. The clustering of the external MOC samples resulted in 13 MOC^{K1} and 8 MOC^{K2} (Figure 6A). Due to the

(D) Confusion matrix showing step 1 LR classification results.
(E) Comparison of step 2 accuracies (LR versus SVM) across EOM, pMC, other lesions, and normal ovary.
(F) Confusion matrix showing step 2 SVM classification results.
(G) Confusion matrix showing combined step 1 (LR) and step 2 (SVM) classification results.
(H) t-SNE representation of DNA methylation profiles (as in Figure 3C) showing MOT and EOM samples classified by the step 1 + step 2 pipeline. Correctly classified samples (green), misclassified samples (red).

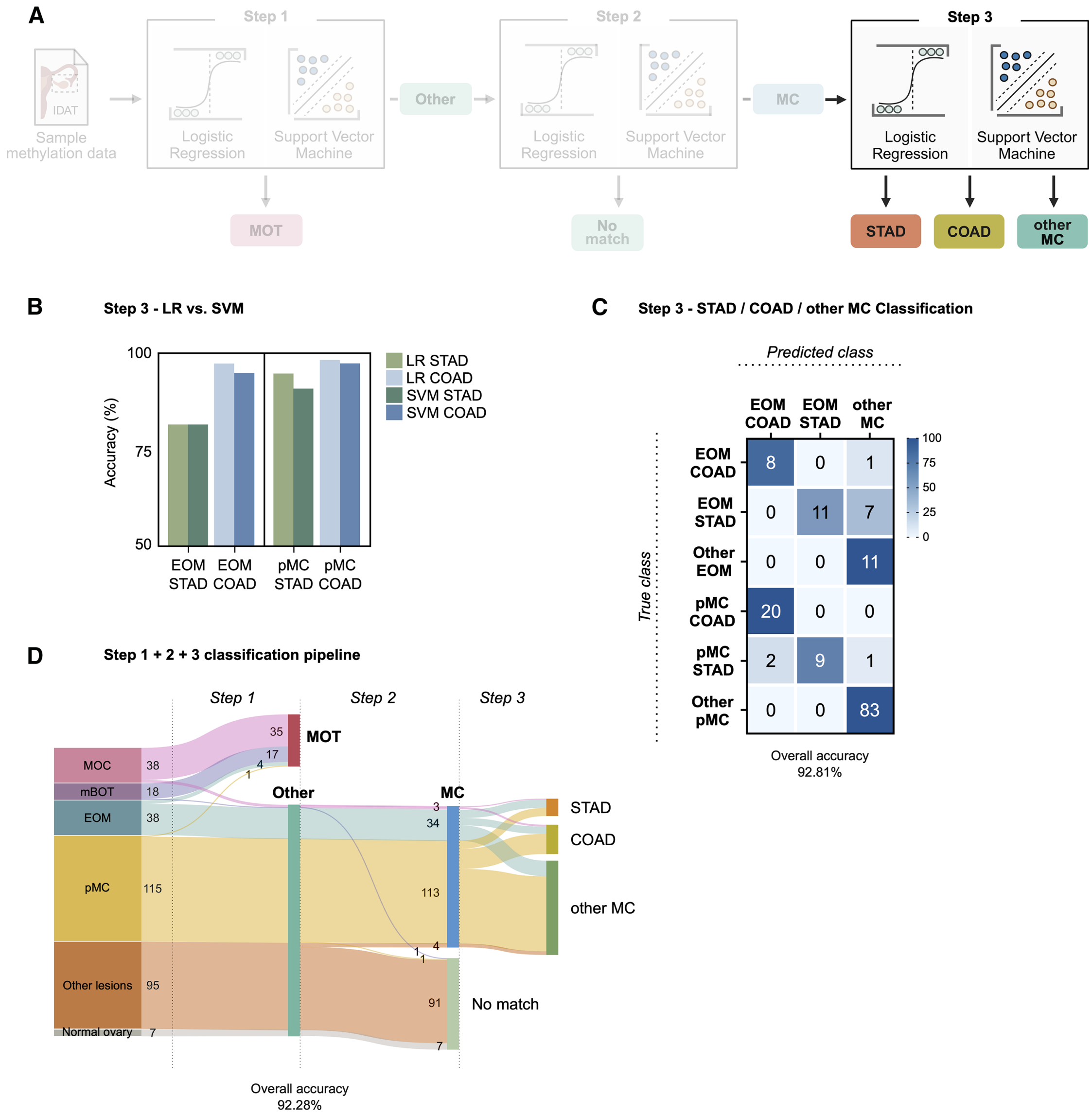

Figure 5. Exploring the origin of EOM

(A) Schematic of step 3 of the DNA-methylation-based classifier, which predicts the tissue of origin for EOM.

(B) Comparison of step 3 classification accuracies using LR and SVM across tumor groups: EOM, pMC, other lesions, and normal ovary.

(C) Confusion matrix showing step 3 LR classification results. EOM and pMC cases are displayed separately.

(D) Sankey plot illustrating per-sample classification flow through steps 1–3 of the pipeline.

recent nature of the external validation cohort, the limited follow-up duration precluded a statistically powered survival analysis. Hence, for the analysis of K1 and K2 MOC subtypes, we performed DMP analysis in MOC^{K1} and MOC^{K2} versus normal ovary, along with CNA profiling and cellular decomposition using the DNA methylation data. The absolute number of DMPs between MOC^{K1} and normal ovary was 46,781 and between MOC^{K2} and normal ovary was 69,647, while only four DMPs were identified between MOC^{K1} and MOC^{K2}, indicating minimal differences between the two subtypes (Figure 6E). This is in line with the internal cohort where we observed a smaller number of DMPs between MOC^{K1} and normal ovary than between MOC^{K2}. CNA analysis showed the same trends as in the internal set, with MOC^{K1} revealing higher levels of *TP53* losses (76.9% versus 25%) and *ERBB2* gains (61.53% versus 12.5%) compared to MOC^{K2} (Figure S6C). As in the internal cohort, cancer cell proportions were significantly higher in MOC^{K2} compared to MOC^{K1} (unpaired *t* test, $p = 0.0093$) (Figure 6F), while fibroblast proportions were higher, but not statistically significant in MOC^{K1} compared to MOC^{K2} (Figure S6D). Regarding the $CD8^{+}$ T cells proportions, no differences were observed between the two groups; this could be explained in part by the much lower sample number (Figure S6E). Statistical comparisons between MOC^{K1} and MOC^{K2} were non-significant for most available

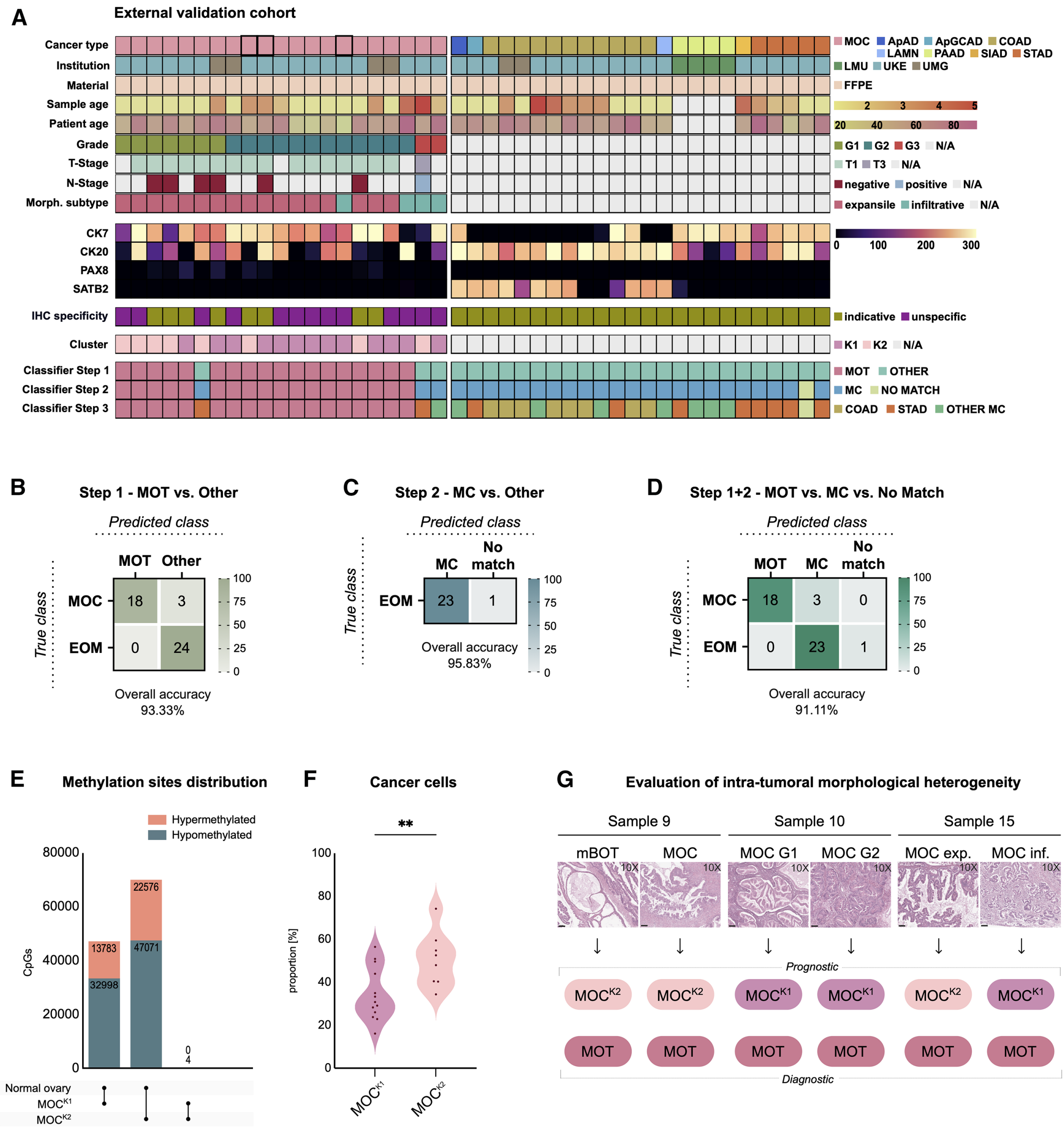

Figure 6. External validation of the classifier and DNA methylation subtypes of MOC

(A) Clinicopathological, immunohistochemical, and classification and subclassification features of the external validation cohort. MOC samples that were analyzed twice are marked with bold rectangles.

(B) Confusion matrix showing step 1 classification results in the external validation cohort.

(C) Confusion matrix showing step 2 classification results in the external validation cohort.

(D) Confusion matrix showing combined step 1 + 2 classification results in the external validation cohort.

(E) Distribution of DMPs identified in pairwise comparisons among normal ovary, MOC^{K1} in the external validation cohort (n = 13), and MOC^{K2} in the external validation cohort (n = 8).

Bars show numbers of hypermethylated (orange) and hypomethylated (green) CpGs.

(F) Inferred fractions (%) of cancer cells across MOC^{K1} and MOC^{K2} in the external validation cohort, with normal ovary as reference. Statistical comparisons were made using unpaired t test. $^{**}p < 0.01$.

(G) Evaluation of intra-tumoral morphological heterogeneity regarding DNA-methylation-based prognostic and diagnostic classifications.

Spatial analysis of two histologically distinct regions from three representative cases: sample 9: mBOT and invasive MOC; sample 10: G1 and G2 MOC; sample 15: expansive and infiltrative growth patterns (magnification, ×10; scale bars, 100 μm).

CellPress
OPEN ACCESS

parameters and showed borderline significance for grade (Fisher exact test, $p = 0.0442$) (Table S7).

Because MOCs are very heterogeneous tumors, we wanted to see if different morphologies impose diagnosis and stratification problems. We selected three heterogeneous MOC samples and microdissected spatially distinct tumor regions (mBOT versus MOC, G1 versus G2, and expansile versus infiltrative growth), which were analyzed separately. For the diagnosis analysis, all six regions were correctly classified as MOT (Figure 6G). However, the stratification into MOC^{K1} and MOC^{K2} showed some degree of heterogeneity. In one sample, the region with infiltrative growth was assigned MOC^{K1}, whereas the region with expansile growth was assigned MOC^{K2}. The other two samples showed concordant subclassification across the analyzed regions (Figure 6G).

DISCUSSION

In this study, we address two important aspects of MOCs: their stratification and relationship to mBOTs, as well as the differential diagnosis with EOMs. We believe these aspects hold significant clinical relevance. To explore these topics, we employed DNA methylation profiling on a large cohort of MOCs, mBOTs, and EOMs samples. Genome-wide DNA methylation analysis has previously been applied to smaller MOC datasets within the broader context of ovarian carcinomas, demonstrating that MOCs represent a distinct ovarian carcinoma subclass.[3]

Our findings provide further evidence for the evolutionary relationship between mBOTs and MOCs, demonstrating a close spatial relationship in the DNA methylation t-SNE analysis between mBOTs and MOCs and important similarities in the DNA methylation relatedness network. Furthermore, we observe two epigenetic patterns of mBOT: one type closer to normal tissue ($mBOT^{normal\_like}$) and another type closer to MOC ($mBOT^{MOC\_like}$), both exhibiting specific spatial relationships and epigenomic relatedness with these tissue types. We made a similar observation in our previous study, which analyzed the epigenetic relationship of MCN to MOT and noted that some mBOT samples tend to share DNA methylation similarities with MCN high-grade/invasive tumors and MOCs, while others tend to resemble MCN low-grade tumors.[7] This suggests that the $mBOT^{MOC\_like}$ could represent a more advanced intraepithelial stage. However, as our study does not include the full spectrum of precursors (benign cystadenomas, Brenner-tumor-associated lesions, and teratoma-associated lesions), these results should be interpreted as supportive of a stepwise transformation model rather than a definitive conclusion. To confirm this hypothesis, a larger cohort with longer follow-up is needed. Such studies will be essential to determine if these molecular signatures have clinical utility, such as avoiding overly conservative surgery like unilateral salpingo-oophorectomy,[39,40] potentially adding therapy for the $mBOT^{MOC\_like}$ subgroup to prevent recurrence[39,40] or increasing surveillance measures.

Similarly, the DNA methylation analysis characterized two distinct types of MOC, which showed differing survival associations in the internal cohort, with MOC^{K2} exhibiting more aggressive outcomes. Except for the T-stage, no other parameter demonstrated prognostic value, further supporting the notion that grading lacks prognostic power for MOC.[21] The clinical course of MOC is highly variable; some cases exhibit aggressive progression, while most remain indolent.[41] Consequently, there is a need for molecular markers to better understand patient risk. If validated in larger, prospective studies, such molecular stratification could help identify the patients who may require more intensive treatment, such as the addition of targeted therapies, including the evaluation of emerging therapies like *KRAS*-inhibitors.[42] Furthermore, our characterization of MOC identifies distinct molecular features that may warrant further investigation into personalized treatment strategies. For instance, the higher levels of T-lymphocytes observed in MOC^{K1} could suggest a rationale for exploring immune checkpoint inhibition. Similarly, MOC^{K1} samples harboring *ERBB2* gain might represent a population for investigating HER2 inhibitors/antibody-drug conjugates.[42] On the other hand, MOC^{K2} cases, with more frequent *CCNE1* gains, suggest these tumors could be evaluated for sensitivity to cyclin E1 pathway inhibitors.[43,44] However, given the observational nature of our study, these associations remain speculative and require functional validation to determine their relevance.

The second aspect of MOC addressed in this study is its differential diagnosis from EOM. We have employed DNA methylation profiles, combined with machine learning, to tackle this challenge. We developed a three-step DNA methylation classifier: in the first step, it identifies MOC; in the second step, it detects EOM and filters out other lesions through an anomaly detection layer; and in the final step, it determines the origin of EOM. Currently, the final step can differentiate between STAD, COAD, and other pMCs. We plan to expand this step to incorporate additional origins of EOM. The clinical relevance of our classifier is significant, as the diagnosis of an MOT often requires a labor-intensive process to exclude the possibility of an occult primary mucinous carcinoma from other sites.[12,45] The classifier is available for other clinicians online at https://app.epiresolve.com/.

Our classifier achieves an accuracy of 95.5% (internal validation) and 91.11% (external validation) in detecting MOC and EOM. These results are generally superior to previous IHC and molecular-based classifiers. Previous studies that focused on morphology and IHC for differentiating MOCs from EOMs report accuracy ranges from 71.3% to 96.1%,[22,46] with only one study surpassing the accuracy of our classifier.[47] In that study, the diagnosis was based on tumor size, bilaterality, and the presence of surface nodules, although its high accuracy was not confirmed by other groups. The mutations in MOC and EOM are similar, with only *APC* and *SMAD4* mutations potentially contributing to the diagnostic algorithm, both suggesting EOM.[3] Transcriptomic studies focusing on the differential diagnosis between MOCs and EOMs are limited and largely unsuccessful. For example, a 19-gene panel failed to distinguish MOCs from EOMs.[48] An alternative approach to improve the diagnosis of MOC versus EOM involves digital pathology. For instance, Wu et al. developed an artificial intelligence (AI) tool to differentiate ovarian carcinoma subtypes using cytological images, achieving an accuracy of 78.2%.[49]

Furthermore, we explored the potential of predicting the origin of the EOMs using DNA methylation data. The classification

accuracy was lower compared to steps 1 and 2: 78.95% (internal cohort) and 73.91% (external cohort). Given the current limitations in the diversity of the other MC category, which currently lacks rare mimics such as gastric-type endocervical adenocarcinoma and specific pancreatobiliary neoplasms, we consider this analysis preliminary and not ready for clinical implementation. By contrast, the primary objective of this diagnostic framework is the robust differentiation of MOC from EOM, where the classifier achieves high diagnostic performance.

We explored moving the diagnosis process into the intraoperative setting. Nanopore technology is advancing as a reliable tool for performing intraoperative DNA methylation classification of cancers.[38,50] In this context, our initial results were suboptimal. The preliminary data indicated that the issue was not the lower number of CpG probes obtained by Nanopore sequencing, but rather the tumor purity of FF samples, which were collected blindly during surgery. Therefore, our findings suggest that intraoperative DNA methylation diagnosis remains an open problem and will require strategies that ensure better sampling.

In conclusion, using genome-wide DNA methylation profiling, we were able to subclassify mBOTs and MOCs, identifying two MOC subtypes with prognostic implications. Furthermore, these two MOC subtypes exhibit distinct molecular characteristics and tumor microenvironment changes, which could have clinical relevance. Finally, we developed a DNA methylation classifier that can differentiate MOC from EOM, potentially enhancing and accelerating the diagnostic workflow for ovarian mucinous lesions.

Limitations of the study

Moreover, it is important to acknowledge several limitations. First, we did not validate the prognostically relevant observations of the MOC subclassification because of the limited follow-up duration of these recently diagnosed samples. Second, our classifier can pinpoint only two origins of EOM, and rare metastases to the ovary are all grouped together as "other." In addition, the classifier has not been tested against rare but diagnostically critical mimics such as gastric-type endocervical adenocarcinoma. Consequently, step 3 should be regarded as a proof-of-concept analysis. The main purpose of our classifier is to differentiate MOTs from EOMs, and addressing the origin of adenocarcinoma metastases will represent a future work. Third, our study is primarily based on DNA methylation data. It would be valuable to incorporate additional omics technologies to further refine the subclassification of MOCs. Fourth, we did not investigate the full spectrum of MOC precursors, such as Brenner tumors, teratomas, and mucinous cystadenomas. This limitation arises partly because DNA methylation analysis requires large tissue samples, and alternative methods, such as spatially resolved proteomics,[51] are needed for such analyses. Future work could also integrate the ovarian surface epithelium and Walthard cell nests for a more comprehensive analysis.

RESOURCE AVAILABILITY

Lead contact

Further information and requests for resources and reagents should be directed to and will be fulfilled by the lead contact, Mihnea P. Dragomir (mihnea.dragomir@charite.de).

Materials availability

This study did not generate new unique reagents.

Data and code availability

- DNA methylation data generated in this study are deposited in the Gene Expression Omnibus (GEO) under accession number GEO: GSE310580.
- The machine learning classifier code and analysis scripts have been deposited at GitHub (https://github.com/teocalina/moc-eom-classifier-model) and are publicly available at https://doi.org/10.5281/zenodo.20408260.
- Any additional data reported in this work is available from the lead contact upon reasonable request.

ACKNOWLEDGMENTS

We acknowledge the technical assistance of Daniel Teichmann and Sandra Meier (Institute for Neuropathology, Charité-Universitätsmedizin Berlin) and Barbara Meyer-Bartell and Kerstin Witkowski (Institute of Pathology Charité-Universitätsmedizin Berlin). We acknowledge the support of the staff of the archive of Institute of Pathology Charité-Universitätsmedizin Berlin and the Edinburgh Bioresource for assistance with sample retrieval. We thank Anna Trelinska-Finger for providing clinical data. The work of M.P.D. was supported by the Berlin Institute of Health, Clinician Scientist Program; DKTK Berlin Young Investigator Grant 2022; an Else Kroner-Fresenius Foundation First and Second Applications Grant 2024_EKEA.77; and Berliner Krebsgesellschaft (DRFF202204). C.G. and M.C. are supported by the Nicola Murray Foundation. The Australian Ovarian Cancer Study (AOCS) Group was supported by the U.S. Army Medical Research and Materiel Command (DAMD17-01-1-0729), The Cancer Council Victoria, Queensland Cancer Fund, The Cancer Council New South Wales, The Cancer Council South Australia, The Cancer Council Tasmania and The Cancer Foundation of Western Australia (Multi-State Applications 191, 211, and 182), and the National Health and Medical Research Council of Australia (NHMRC; ID199600, ID400413, and ID400281). The GAMuT study was funded by the NHMRC (ID1045783). The AOCS acknowledges support from Ovarian Cancer Australia and the Peter MacCallum Foundation. The AOCS acknowledges the cooperation of the participating institutions in Australia and acknowledges the contribution of the study nurses, research assistants, and clinical and scientific collaborators to the study. The complete AOCS Study Group can be found at www.aocstudy.org. We would like to thank all of the women who participated in these research programs.

AUTHOR CONTRIBUTIONS

T.G.C. and E.G. performed investigation, software development, formal analysis, validation, and methodology and wrote the original draft. M.M. provided resources, contributed to methodology and validation, and reviewed and edited the manuscript. B.D., A.A.S., I.K., and T.J. contributed to investigation, methodology, and manuscript review. S.S., B.C., M.C., C.G., E.L.C., C.V., V.H., W.D.S., N.L., F.H., A.V., Y.T., F.B., F.K., R.B., P.S., E.B., G.S., F.L.H., E.T.T., J.S., and I.E.B. provided resources, validation, and data curation and contributed to review and editing. P.E. contributed to investigation, methodology, data curation, and resources and reviewed the manuscript. D.H., K.L.G., and D.C. provided resources, supervision, funding acquisition, and methodology and contributed to review and editing. M.P.D. conceived and supervised the study, acquired funding, validated data, and co-wrote the manuscript. All authors approved the final version of the manuscript.

DECLARATION OF INTERESTS

D.C. is co-founder and shareholder of Heidelberg Epignostix GmbH. F.K. is lead medical advisor and board member of Aignostics. S.S. is a part-time employee at Aignostics. D.H. is a member of the scientific advisory board at Aignostics.

DECLARATION OF GENERATIVE AI AND AI-ASSISTED TECHNOLOGIES IN THE WRITING PROCESS

During the preparation of this manuscript, the authors used ChatGPT-5, Gemini 3, and Claude 4.6 to assist with readability and text editing. After using these tools, the authors reviewed and edited the content as needed and take full responsibility for the content of the published article.

STAR★METHODS

Detailed methods are provided in the online version of this paper and include the following:

- KEY RESOURCES TABLE
- EXPERIMENTAL MODEL AND STUDY PARTICIPANT DETAILS
- METHOD DETAILS
  - Diagnosis review
  - TMA construction
  - Immunohistochemistry staining and quantification, IHC classification
  - DNA extraction
  - DNA methylation
  - DNA panel sequencing and mutation call
  - DNA methylation array processing
  - Tumor purity estimation
  - t-distributed stochastic neighbor embedding (t-SNE) and cluster analysis
  - Copy number analysis
  - Differently methylated CpG probes and pathway analysis
  - Clustering analysis
  - Cell type deconvolution
  - Other DNA methylation sets used for training and testing the classifier
  - Classification pipeline development
  - DNA methylation intratumoral heterogeneity
  - Nanopore low-pass whole genome sequencing
  - Methylation-based classification from sparse methylomes
- QUANTIFICATION AND STATISTICAL ANALYSIS

SUPPLEMENTAL INFORMATION

Supplemental information can be found online at https://doi.org/10.1016/j.xcrm.2026.102941.

Received: November 28, 2025
Revised: April 1, 2026
Accepted: June 29, 2026
Published: July 27, 2026

REFERENCES

1. Cheasley, D., Wakefield, M.J., Ryland, G.L., Allan, P.E., Alsop, K., Amarasinghe, K.C., Ananda, S., Anglesio, M.S., Au-Yeung, G., Böhm, M., et al. (2019). The molecular origin and taxonomy of mucinous ovarian carcinoma. Nat. Commun. *10*, 3935. https://doi.org/10.1038/s41467-019-11862-x.

2. Kurman, R.J., and Shih, I.-M. (2016). The Dualistic Model of Ovarian Carcinogenesis: Revisited, Revised, and Expanded. Am. J. Pathol. *186*, 733–747. https://doi.org/10.1016/j.ajpath.2015.11.011.

3. Meagher, N.S., Köbel, M., Karnezis, A.N., Talhouk, A., Anglesio, M.S., Berchuck, A., Gayther, S.A., Pharoah, P.P., Webb, P.M., Ramus, S.J., and Gorringe, K.L. (2025). Cellular origins of mucinous ovarian carcinoma. J. Pathol. *266*, 9–25. https://doi.org/10.1002/path.6407.

4. Seidman, J.D., and Krishnan, J. (2017). Ovarian Epithelial Inclusions With Mucinous Differentiation: A Clinicopathologic Study of 42 Cases. Int. J. Gynecol. Pathol. *36*, 372–376. https://doi.org/10.1097/PGP.0000000000000348.

5. Simons, M., Simmer, F., Bulten, J., Ligtenberg, M.J., Hollema, H., van Vliet, S., de Voer, R.M., Kamping, E.J., van Essen, D.F., Ylstra, B., et al. (2020). Two types of primary mucinous ovarian tumors can be distinguished based on their origin. Mod. Pathol. *33*, 722–733. https://doi.org/10.1038/s41379-019-0401-y.

6. Seidman, J.D., and Khedmati, F. (2008). Exploring the histogenesis of ovarian mucinous and transitional cell (Brenner) neoplasms and their relationship with Walthard cell nests: a study of 120 tumors. Arch. Pathol. Lab Med. *132*, 1753–1760. https://doi.org/10.5858/132.11.1753.

7. Leoni, Z., Calina, T.G., Janik, T., Grafenhorst, E., Taube, E.T., Neumann, C.C., Chen, B., Braicu, E.I., Sehouli, J., Malinka, T., et al. (2025). Mucinous cystic neoplasms of the pancreas and liver share a similar DNA methylation profile with mucinous ovarian tumors. J. Pathol. *267*, 10–24. https://doi.org/10.1002/path.6439.

8. Kubeček, O., Laco, J., Špaček, J., Petera, J., Kopecký, J., Kubečková, A., and Filip, S. (2017). The pathogenesis, diagnosis, and management of metastatic tumors to the ovary: a comprehensive review. Clin. Exp. Metastasis *34*, 295–307. https://doi.org/10.1007/s10585-017-9856-8.

9. Bruls, J., Simons, M., Overbeek, L.I., Bulten, J., Massuger, L.F., and Nagtegaal, I.D. (2015). A national population-based study provides insight in the origin of malignancies metastatic to the ovary. Virchows Arch. *467*, 79–86. https://doi.org/10.1007/s00428-015-1771-2.

10. Alvarado-Cabrero, I., Rodríguez-Gómez, A., Castelan-Pedraza, J., and Valencia-Cedillo, R. (2013). Metastatic ovarian tumors: a clinicopathologic study of 150 cases. Anal. Quant. Cytopathol. Histpathol. *35*, 241–248.

11. Morice, P., Gouy, S., and Leary, A. (2019). Mucinous Ovarian Carcinoma. N. Engl. J. Med. *380*, 1256–1266. https://doi.org/10.1056/NEJMra1813254.

12. Seidman, J.D., Kurman, R.J., and Ronnett, B.M. (2003). Primary and metastatic mucinous adenocarcinomas in the ovaries: incidence in routine practice with a new approach to improve intraoperative diagnosis. Am. J. Surg. Pathol. *27*, 985–993. https://doi.org/10.1097/00000478-200307000-00014.

13. Lee, K.R., and Young, R.H. (2003). The distinction between primary and metastatic mucinous carcinomas of the ovary: gross and histologic findings in 50 cases. Am. J. Surg. Pathol. *27*, 281–292. https://doi.org/10.1097/00000478-200303000-00001.

14. Carlson, J.W., Gilks, C.B., and Soslow, R.A. (2023). Tumors of the Ovary and Fallopian Tube (American Registry of Pathology)).

15. Capper, D., Jones, D.T.W., Sill, M., Hovestadt, V., Schrimpf, D., Sturm, D., Koelsche, C., Sahm, F., Chavez, L., Reuss, D.E., et al. (2018). DNA methylation-based classification of central nervous system tumours. Nature *555*, 469–474. https://doi.org/10.1038/nature26000.

16. Moran, S., Martínez-Cardús, A., Sayols, S., Musulén, E., Balañá, C., Estival-Gonzalez, A., Moutinho, C., Heyn, H., Diaz-Lagares, A., de Moura, M.C., et al. (2016). Epigenetic profiling to classify cancer of unknown primary: a multicentre, retrospective analysis. Lancet Oncol. *17*, 1386–1395. https://doi.org/10.1016/S1470-2045(16)30297-2.

17. Calina, T.G., Perez, E., Grafenhorst, E., Benhamida, J., Schallenberg, S., Popescu, A., Koch, I., Janik, T., Chen, B., Ihlow, J., et al. (2024). DNA methylation classifier to diagnose pancreatic ductal adenocarcinoma metastases from different anatomical sites. Clin. Epigenetics *16*, 156. https://doi.org/10.1186/s13148-024-01768-x.

18. Dragomir, M.P., Calina, T.G., Perez, E., Schallenberg, S., Chen, M., Albrecht, T., Koch, I., Wolkenstein, P., Goeppert, B., Roessler, S., et al. (2023). DNA methylation-based classifier differentiates intrahepatic pancreato-biliary tumours. EBioMedicine *93*, 104657. https://doi.org/10.1016/j.ebiom.2023.104657.

19. Jurmeister, P., Bockmayr, M., Seegerer, P., Bockmayr, T., Treue, D., Montavon, G., Vollbrecht, C., Arnold, A., Teichmann, D., Bressem, K., et al. (2019). Machine learning analysis of DNA methylation profiles distinguishes primary lung squamous cell carcinomas from head and neck

metastases. Sci. Transl. Med. *11*, eaaw8513. https://doi.org/10.1126/scitranslmed.aaw8513.

20. Lee, K.R., and Scully, R.E. (2000). Mucinous tumors of the ovary: a clinicopathologic study of 196 borderline tumors (of intestinal type) and carcinomas, including an evaluation of 11 cases with "pseudomyxoma peritonei.". Am. J. Surg. Pathol. *24*, 1447–1464. https://doi.org/10.1097/00000478-200011000-00001.
21. Organisation mondiale de la santé and Centre international de recherche sur le cancer (2014). WHO classification of tumours of female reproductive organs. International agency for research on cancer *6*.
22. Dundr, P., Singh, N., Nožičková, B., Němejcová, K., Bártů, M., and Stružinská, I. (2021). Primary mucinous ovarian tumors vs. ovarian metastases from gastrointestinal tract, pancreas and biliary tree: a review of current problematics. Diagn. Pathol. *16*, 20. https://doi.org/10.1186/s13000-021-01079-2.
23. Aldaoud, N., Erashdi, M., AlKhatib, S., Abdo, N., Al-Mohtaseb, A., and Graboski-Bauer, A. (2019). The utility of PAX8 and SATB2 immunohistochemical stains in distinguishing ovarian mucinous neoplasms from colonic and appendiceal mucinous neoplasm. BMC Res. Notes *12*, 770. https://doi.org/10.1186/s13104-019-4816-9.
24. Chai, H.-J., Ren, Q., Fan, Q., Ye, L., Du, G.-Y., Du, H.-W., Xu, W., Li, Y., Zhang, L., and Cheng, Z.-P. (2017). PAX8 is a potential marker for the diagnosis of primary epithelial ovarian cancer. Oncol. Lett. *14*, 5871–5875. https://doi.org/10.3892/ol.2017.6949.
25. Meagher, N.S., Wang, L., Rambau, P.F., Intermaggio, M.P., Huntsman, D.G., Wilkens, L.R., El-Bahrawy, M.A., Ness, R.B., Odunsi, K., Steed, H., et al. (2019). A combination of the immunohistochemical markers CK7 and SATB2 is highly sensitive and specific for distinguishing primary ovarian mucinous tumors from colorectal and appendiceal metastases. Mod. Pathol. *32*, 1834–1846. https://doi.org/10.1038/s41379-019-0302-0.
26. Mueller, J.J., Schlappe, B.A., Kumar, R., Olvera, N., Dao, F., Abu-Rustum, N., Aghajanian, C., DeLair, D., Hussein, Y.R., Soslow, R.A., et al. (2018). Massively parallel sequencing analysis of mucinous ovarian carcinomas: genomic profiling and differential diagnoses. Gynecol. Oncol. *150*, 127–135. https://doi.org/10.1016/j.ygyno.2018.05.008.
27. Mackenzie, R., Kommoss, S., Winterhoff, B.J., Kipp, B.R., Garcia, J.J., Voss, J., Halling, K., Karnezis, A., Senz, J., Yang, W., et al. (2015). Targeted deep sequencing of mucinous ovarian tumors reveals multiple overlapping RAS-pathway activating mutations in borderline and cancerous neoplasms. BMC Cancer *15*, 415. https://doi.org/10.1186/s12885-015-1421-8.
28. Ryland, G.L., Hunter, S.M., Doyle, M.A., Caramia, F., Li, J., Rowley, S.M., Christie, M., Allan, P.E., Stephens, A.N., Bowtell, D.D.L., et al. (2015). Mutational landscape of mucinous ovarian carcinoma and its neoplastic precursors. Genome Med. *7*, 87. https://doi.org/10.1186/s13073-015-0210-y.
29. Bodelon, C., Killian, J.K., Sampson, J.N., Anderson, W.F., Matsuno, R., Brinton, L.A., Lissowska, J., Anglesio, M.S., Bowtell, D.D.L., Doherty, J.A., et al. (2019). Molecular Classification of Epithelial Ovarian Cancer Based on Methylation Profiling: Evidence for Survival Heterogeneity. Clin. Cancer Res. *25*, 5937–5946. https://doi.org/10.1158/1078-0432.CCR-18-3720.
30. Borella, F., Mitidieri, M., Cosma, S., Benedetto, C., Bertero, L., Fucina, S., Ray-Coquard, I., Carapezzi, A., and Ferraioli, D. (2023). Update on Prognostic and Predictive Markers in Mucinous Ovarian Cancer. Cancers *15*, 1172. https://doi.org/10.3390/cancers15041172.
31. Vitiazeva, V., Kattla, J.J., Flowers, S.A., Lindén, S.K., Premaratne, P., Weijdegård, B., Sundfeldt, K., and Karlsson, N.G. (2015). The O-Linked Glycome and Blood Group Antigens ABO on Mucin-Type Glycoproteins in Mucinous and Serous Epithelial Ovarian Tumors. PLoS One *10*, e0130197. https://doi.org/10.1371/journal.pone.0130197.
32. Engqvist, H., Parris, T.Z., Kovács, A., Nemes, S., Werner Rönnerman, E., De Lara, S., Biermann, J., Sundfeldt, K., Karlsson, P., and Helou, K. (2019). Immunohistochemical validation of COL3A1, GPR158 and PITHD1 as prognostic biomarkers in early-stage ovarian carcinomas. BMC Cancer *19*, 928. https://doi.org/10.1186/s12885-019-6084-4.
33. Chang, K.-L., Lee, M.-Y., Chao, W.-R., and Han, C.-P. (2016). The status of Her2 amplification and Kras mutations in mucinous ovarian carcinoma. Hum. Genomics *10*, 40. https://doi.org/10.1186/s40246-016-0096-9.
34. Xi, Q., Kunita, A., Ogawa, M., Ka, M., Tanimoto, S., Tsuchimochi, S., Nagai, S., Matsunaga, A., Fukuda, T., Watanabe, K., et al. (2025). Cyclin E1 overexpression sensitizes ovarian cancer cells to WEE1 and PLK1 inhibition. Oncogene *44*, 1375–1386. https://doi.org/10.1038/s41388-025-03312-4.
35. Biskup, E., Lopacinska-Jørgensen, J., Vestergaard, L.K., and Høgdall, E. (2024). Validating reference-based algorithms to determine cell-type heterogeneity in ovarian cancer DNA methylation studies. Sci. Rep. *14*, 11048. https://doi.org/10.1038/s41598-024-61857-y.
36. Chakravarthy, A., Furness, A., Joshi, K., Ghorani, E., Ford, K., Ward, M.J., King, E.V., Lechner, M., Marafioti, T., Quezada, S.A., et al. (2018). Pan-cancer deconvolution of tumour composition using DNA methylation. Nat. Commun. *9*, 3220. https://doi.org/10.1038/s41467-018-05570-1.
37. Goode, E.L., Block, M.S., Kalli, K.R., Vierkant, R.A., Chen, W., Fogarty, Z.C., Gentry-Maharaj, A., Tołoczko, A., and Hein, A. (2017). Dose-Response Association of CD8+ Tumor-Infiltrating Lymphocytes and Survival Time in High-Grade Serous Ovarian Cancer. JAMA Oncol. *3*, e173290. https://doi.org/10.1001/jamaoncol.2017.3290.
38. Yuan, D., Jugas, R., Pokorna, P., Sterba, J., Slaby, O., Schmid, S., Siewert, C., Osberg, B., Capper, D., Halldorsson, S., et al. (2025). crossNN is an explainable framework for cross-platform DNA methylation-based classification of tumors. Nat. Cancer *6*, 1283–1294. https://doi.org/10.1038/s43018-025-00976-5.
39. Sun, L., Li, N., Song, Y., Wang, G., Zhao, Z., and Wu, L. (2018). Clinicopathologic Features and Risk Factors for Recurrence of Mucinous Borderline Ovarian Tumors: A Retrospective Study With Follow-up of More Than 10 Years. Int. J. Gynecol. Cancer *28*, 1643–1649. https://doi.org/10.1097/IGC.0000000000001362.
40. Della Corte, L., Mercorio, A., Serafino, P., Viciglione, F., Palumbo, M., De Angelis, M.C., Borgo, M., Buonfantino, C., Tesorone, M., Bifulco, G., and Giampaolino, P. (2022). The challenging management of borderline ovarian tumors (BOTs) in women of childbearing age. Front. Surg. *9*, 973034. https://doi.org/10.3389/fsurg.2022.973034.
41. Zaino, R.J., Brady, M.F., Lele, S.M., Michael, H., Greer, B., and Bookman, M.A. (2011). Advanced stage mucinous adenocarcinoma of the ovary is both rare and highly lethal: a Gynecologic Oncology Group study. Cancer *117*, 554–562. https://doi.org/10.1002/cncr.25460.
42. Przywara, G., Biegańska, O., Biczak, E., Białoń, A., Fidorowicz, D., Dankowska, A., Łapińska, Z., and Kulbacka, J. (2025). Recent Therapies and Biomarkers in Mucinous Ovarian Carcinoma. Cells *14*, 1232. https://doi.org/10.3390/cells14161232.
43. Leijen, S., Van Geel, R.M.J.M., Sonke, G.S., De Jong, D., Rosenberg, E.H., Marchetti, S., Pluim, D., Van Werkhoven, E., Rose, S., Lee, M.A., et al. (2016). Phase II Study of WEE1 Inhibitor AZD1775 Plus Carboplatin in Patients With *TP53* -Mutated Ovarian Cancer Refractory or Resistant to First-Line Therapy Within 3 Months. J. Clin. Oncol. *34*, 4354–4361. https://doi.org/10.1200/JCO.2016.67.5942.
44. Kanska, J., Zakhour, M., Taylor-Harding, B., Karlan, B.Y., and Wiedemeyer, W.R. (2016). Cyclin E as a potential therapeutic target in high grade serous ovarian cancer. Gynecol. Oncol. *143*, 152–158. https://doi.org/10.1016/j.ygyno.2016.07.111.
45. Frumovitz, M., Schmeler, K.M., Malpica, A., Sood, A.K., and Gershenson, D.M. (2010). Unmasking the complexities of mucinous ovarian carcinoma. Gynecol. Oncol. *117*, 491–496. https://doi.org/10.1016/j.ygyno.2010.02.010.
46. De Boeck, A., Kang, E.Y., Meagher, N.S., Gorringe, K.L., Lee, C.H., Minoo, P., and Köbel, M. (2026). A 5-marker immunohistochemical panel of CK17, MEP1A, PAX8, SMAD4, and CDX2 to distinguish ovarian mucinous

CellPress
OPEN ACCESS

carcinoma from metastatic pancreatic ductal adenocarcinoma. Histopathology *88*, 402–413. https://doi.org/10.1111/his.70002.

47. Hu, J., Khalifa, R.D., Roma, A.A., and Fadare, O. (2018). The pathologic distinction of primary and metastatic mucinous tumors involving the ovary: A re-evaluation of algorithms based on gross features. Ann. Diagn. Pathol. *37*, 1–6. https://doi.org/10.1016/j.anndiagpath.2018.07.001.

48. Meagher, N.S., Gorringe, K.L., Wakefield, M., Bolithon, A., Pang, C.N.I., Chiu, D.S., Anglesio, M.S., Mallitt, K.-A., Doherty, J.A., Harris, H.R., et al. (2022). Gene-Expression Profiling of Mucinous Ovarian Tumors and Comparison with Upper and Lower Gastrointestinal Tumors Identifies Markers Associated with Adverse Outcomes. Clin. Cancer Res. *28*, 5383–5395. https://doi.org/10.1158/1078-0432.CCR-22-1206.

49. Wu, M., Yan, C., Liu, H., and Liu, Q. (2018). Automatic classification of ovarian cancer types from cytological images using deep convolutional neural networks. Biosci. Rep. *38*, BSR20180289. https://doi.org/10.1042/BSR20180289.

50. Vermeulen, C., Pagès-Gallego, M., Kester, L., Kranendonk, M.E.G., Wesseling, P., Verburg, N., de Witt Hamer, P., Kooi, E.J., Dankmeijer, L., van der Lugt, J., et al. (2023). Ultra-fast deep-learned CNS tumour classification during surgery. Nature *622*, 842–849. https://doi.org/10.1038/s41586-023-06615-2.

51. Makhmut, A., Dragomir, M.P., Fritzsche, S., Moebs, M., Schmitt, W.D., Taube, E.T., and Coscia, F. (2026). Spatial proteomics of ovarian cancer precursors delineates early disease changes and drug targets. Mol Syst Biol. *22*, 7–41. https://doi.org/10.1038/s44320-025-00168-4.

52. Tian, Y., Morris, T.J., Webster, A.P., Yang, Z., Beck, S., Feber, A., and Teschendorff, A.E. (2017). ChAMP: updated methylation analysis pipeline for Illumina BeadChips. Bioinformatics *33*, 3982–3984. https://doi.org/10.1093/bioinformatics/btx513.

53. Daenekas, B., Pérez, E., Boniolo, F., Stefan, S., Benfatto, S., Sill, M., Sturm, D., Jones, D.T.W., Capper, D., Zapatka, M., and Hovestadt, V. (2024). Conumee 2.0: enhanced copy-number variation analysis from DNA methylation arrays for humans and mice. Bioinformatics *40*, btae029. https://doi.org/10.1093/bioinformatics/btae029.

54. van der Maaten, L., and Hinton, G. (2008). Visualizing Data using t-SNE. J. Mach. Learn. Res. *9*, 2579–2605.

55. Zhou, W., Triche, T.J., Laird, P.W., and Shen, H. (2018). SeSAMe: reducing artifactual detection of DNA methylation by Infinium BeadChips in genomic deletions. Nucleic Acids Res. *46*, e123. https://doi.org/10.1093/nar/gky691.

56. Zheng, X., Zhang, N., Wu, H.-J., and Wu, H. (2017). Estimating and accounting for tumor purity in the analysis of DNA methylation data from cancer studies. Genome Biol. *18*, 17. https://doi.org/10.1186/s13059-016-1143-5.

57. Chen, B.H., and Zhou, W. (2024). mLiftOver: harmonizing data across Infinium DNA methylation platforms. Bioinformatics *40*, btae423. https://doi.org/10.1093/bioinformatics/btae423.

58. Bankhead, P., Loughrey, M.B., Fernández, J.A., Dombrowski, Y., McArt, D.G., Dunne, P.D., McQuaid, S., Gray, R.T., Murray, L.J., Coleman, H.G., et al. (2017). QuPath: Open source software for digital pathology image analysis. Sci. Rep. *7*, 16878. https://doi.org/10.1038/s41598-017-17204-5.

59. Xie, Z., Bailey, A., Kuleshov, M.V., Clarke, D.J.B., Evangelista, J.E., Jenkins, S.L., Lachmann, A., Wojciechowicz, M.L., Kropiwnicki, E., Jagodnik, K.M., et al. (2021). Gene Set Knowledge Discovery with Enrichr. Curr. Protoc. *1*, e90. https://doi.org/10.1002/cpz1.90.

60. Cardillo, N., Devor, E.J., Pedra Nobre, S., Newtson, A., Leslie, K., Bender, D.P., Smith, B.J., Goodheart, M.J., and Gonzalez-Bosquet, J. (2022). Integrated Clinical and Genomic Models to Predict Optimal Cytoreduction in High-Grade Serous Ovarian Cancer. Cancers *14*, 3554. https://doi.org/10.3390/cancers14143554.

61. McCarty, K.S., Szabo, E., Flowers, J.L., Cox, E.B., Leight, G.S., Miller, L., Konrath, J., Soper, J.T., Budwit, D.A., and Creasman, W.T. (1986). Use of a monoclonal anti-estrogen receptor antibody in the immunohistochemical evaluation of human tumors. Cancer Res. *46*, 4244s–4248s.

62. Kim, S.-W., Roh, J., and Park, C.-S. (2016). Immunohistochemistry for Pathologists: Protocols, Pitfalls, and Tips. J. Pathol. Transl. Med. *50*, 411–418. https://doi.org/10.4132/jptm.2016.08.08.

63. Grout, J.A., Sirven, P., Leader, A.M., Maskey, S., Hector, E., Puisieux, I., Steffan, F., Cheng, E., Tung, N., Maurin, M., et al. (2022). Spatial Positioning and Matrix Programs of Cancer-Associated Fibroblasts Promote T-cell Exclusion in Human Lung Tumors. Cancer Discov. *12*, 2606–2625. https://doi.org/10.1158/2159-8290.CD-21-1714.

64. Aryee, M.J., Jaffe, A.E., Corrada-Bravo, H., Ladd-Acosta, C., Feinberg, A.P., Hansen, K.D., and Irizarry, R.A. (2014). Minfi: a flexible and comprehensive Bioconductor package for the analysis of Infinium DNA methylation microarrays. Bioinformatics *30*, 1363–1369. https://doi.org/10.1093/bioinformatics/btu049.

65. Fortin, J.-P., Labbe, A., Lemire, M., Zanke, B.W., Hudson, T.J., Fertig, E.J., Greenwood, C.M., and Hansen, K.D. (2014). Functional normalization of 450k methylation array data improves replication in large cancer studies. Genome Biol. *15*, 503. https://doi.org/10.1186/s13059-014-0503-2.

66. Chen, E.Y., Tan, C.M., Kou, Y., Duan, Q., Wang, Z., Meirelles, G.V., Clark, N.R., and Ma'ayan, A. (2013). Enrichr: interactive and collaborative HTML5 gene list enrichment analysis tool. BMC Bioinf. *14*, 128. https://doi.org/10.1186/1471-2105-14-128.

67. Kuleshov, M.V., Jones, M.R., Rouillard, A.D., Fernandez, N.F., Duan, Q., Wang, Z., Koplev, S., Jenkins, S.L., Jagodnik, K.M., Lachmann, A., et al. (2016). Enrichr: a comprehensive gene set enrichment analysis web server 2016 update. Nucleic Acids Res. *44*, W90–W97. https://doi.org/10.1093/nar/gkw377.

68. Ding, C., and He, X. (2004). *K* -means clustering via principal component analysis. In Twenty-first international conference on Machine learning - ICML '04 (ACM Press), p. 29. https://doi.org/10.1145/1015330.1015408.

69. Bengtsson, H. (2009). matrixStats. Functions that Apply to Rows and Columns of Matrices (and to Vectors). https://doi.org/10.32614/CRAN.package.

70. Wickham, H., Chang, W., Henry, L., Pedersen, T.L., Takahashi, K., Wilke, C., Woo, K., Yutani, H., Dunnington, D., and Van Den Brand, T. (2007). ggplot2: Create Elegant Data Visualisations Using the Grammar of Graphics. Comprehensive R Archive Network (CRAN). https://doi.org/10.32614/CRAN.package.ggplot2.

71. Kolde, R. (2010). Pheatmap: Pretty Heatmaps. Comprehensive R Archive Network (CRAN). https://doi.org/10.32614/CRAN.package.pheatmap.

72. Garcia-Recio, S., Hinoue, T., Wheeler, G.L., Kelly, B.J., Garrido-Castro, A.C., Pascual, T., De Cubas, A.A., Xia, Y., Felsheim, B.M., McClure, M.B., et al. (2023). Multiomics in primary and metastatic breast tumors from the AURORA US network finds microenvironment and epigenetic drivers of metastasis. Nat. Cancer *4*, 128–147. https://doi.org/10.1038/s43018-022-00491-x.

73. Joo, J.E., Mahmood, K., Walker, R., Georgeson, P., Candiloro, I., Clendenning, M., Como, J., Joseland, S., Preston, S., Graversen, L., et al. (2023). Identifying primary and secondary MLH1 epimutation carriers displaying low-level constitutional MLH1 methylation using droplet digital PCR and genome-wide DNA methylation profiling of colorectal cancers. Clin. Epigenetics *15*, 95. https://doi.org/10.1186/s13148-023-01511-y.

74. Morotti, A., Gentile, F., Lopez, G., Passignani, G., Valenti, L., Locatelli, M., Caroli, M., Fanizzi, C., Ferrero, S., and Vaira, V. (2023). Epigenetic Rewiring of Metastatic Cancer to the Brain: Focus on Lung and Colon Cancers. Cancers *15*, 2145. https://doi.org/10.3390/cancers15072145.

75. Hirano, T., Arai, E., Fujimoto, M., Nakayama, Y., Tian, Y., Ito, N., Makabe, T., Yamagami, W., Susumu, N., Aoki, D., and Kanai, Y. (2022). Prognostication of early-onset endometrioid endometrial cancer based on genome-wide DNA methylation profiles. J. Gynecol. Oncol. *33*, e74. https://doi.org/10.3802/jgo.2022.33.e74.

76. Cerapio, J.P., Marchio, A., Cano, L., López, I., Fournié, J.-J., Régnault, B., Casavilca-Zambrano, S., Ruiz, E., Dejean, A., Bertani, S., and Pineau, P. (2021). Global DNA hypermethylation pattern and unique gene expression signature in liver cancer from patients with Indigenous American ancestry. Oncotarget *12*, 475–492. https://doi.org/10.18632/oncotarget.27890.

77. Benhamida, J.K., Vyas, M., Tanaka, A., Wang, L., Bahrami, A., Ozcan, K., Basturk, O., Villafania, L., Mata, D.A., El Jabbour, T., et al. (2022). Pancreatoblastomas and mixed and pure acinar cell carcinomas share epigenetic signatures distinct from other neoplasms of the pancreas. Mod. Pathol. *35*, 956–961. https://doi.org/10.1038/s41379-021-00989-2.

78. Brugger J., Hultschig C., Baumhoer D., Ameline B., Freyter B.M., Frank S., Hench J. Mepylome: A Point-of-Care Tumor Diagnostic Toolkit for Tumor DNA Methylation and Copy Number Analysis. Adv. Intell. Syst. 2025; 000, e202500778. https://doi.org/10.1002/aisy.202500778.

79. Pedregosa, F., Varoquaux, G., Gramfort, A., Michel, V., Thirion, B., Grisel, O., Blondel, M., Prettenhofer, P., Weiss, R., Dubourg, V., et al. (2011). Scikit-learn: Machine Learning in Python. J. Mach. Learn. Res. *12*, 2825–2830.

80. Akiba, T., Sano, S., Yanase, T., Ohta, T., and Koyama, M. (2019). Optuna: A Next-generation Hyperparameter Optimization Framework. In Proceedings of the 25th ACM SIGKDD International Conference on Knowledge Discovery & Data Mining KDD '19 (Association for Computing Machinery)), pp. 2623–2631. https://doi.org/10.1145/3292500.3330701.

81. Kuschel, L.P., Hench, J., Frank, S., Hench, I.B., Girard, E., Blanluet, M., Masliah-Planchon, J., Misch, M., Onken, J., Czabanka, M., et al. (2023). Robust methylation-based classification of brain tumours using nanopore sequencing. Neuropathol. Appl. Neurobiol. *49*, e12856. https://doi.org/10.1111/nan.12856.

82. Li, H. (2018). Minimap2: pairwise alignment for nucleotide sequences. Bioinformatics *34*, 3094–3100. https://doi.org/10.1093/bioinformatics/bty191.

STAR★METHODS

KEY RESOURCES TABLE

REAGENT or RESOURCE	SOURCE	IDENTIFIER
**Antibodies**		
anti-ANXA1	BD Biosciences	Cat# 610067; RRID: AB_397478
anti-CDX2	Zytomed Systems	Cat# RBK019-05, Clone EPR2764T
anti-CK7	Agilent	Cat# M7018; RRID: AB_2134589
anti-ER	Ventana, Roche Diagnostics	Cat# 790-4325; RRID: AB_2335977
anti-MUC5AC	Leica	Cat# 18-2322; RRID: AB_442113
anti-p16	Ventana, Roche Diagnostics	Cat# 805-4713; RRID: AB_3675558
anti-p53	Agilent	Cat# M7001; RRID: AB_2206626
anti-panCK	Agilent	Cat# M0821; RRID: AB_2858276
anti-PAX8	Ventana, Roche Diagnostics	Cat# 760–4618, Clone MRQ-50
anti-PR	Ventana, Roche Diagnostics	Cat# 790–4296; RRID: AB_2335976
anti-SATB2	Abcam	Cat# ab92446; RRID: AB_10563678
anti-SMAD4	Abcam	Cat# ab40759; RRID: AB_777980
anti-CD8	Agilent	Cat# M7103; RRID: AB_2075537
**Critical commercial assays**		
Illumina Infinium MethylationEPIC (EPIC) BeadChip Kit	Illumina	Cat# WG-317-1003
Illumina Infinium MethylationEPIC v2.0 (EPICv2) BeadChip Kit	Illumina	Cat# 20020459
GridION R10.4.1 Flow Cell	Oxford Nanopore Technologies	Cat# FLO-MIN114
Ion AmpliSeq Cancer Hotspot Panel v2	ThermoFisher Scientific	Cat# 4475346
Cell Conditioning1 (CC1)	Ventana, Roche Diagnostics	Cat# 950-224
UltraView Universal DAB Detection Kit	Ventana, Roche Diagnostics	Cat# 760-500
Ventana Benchmark ULTRA automated staining platform	Ventana, Roche Diagnostics	N/A
**Deposited data**		
DNA methylation data	this paper	GEO: GSE310580
**Software and algorithms**		
Classifier Training Code	this paper	https://doi.org/10.5281/zenodo.20408260
R	R package The R Project for Statistical Computing	v4.3.3; https://www.r-project.org/
minfi R package	Bioconductor	v1.21.4; https://bioconductor.org/packages/minfi
limma R package	Bioconductor	v3.30.11; https://bioconductor.org/packages/limma
ChAMP R package	Tian et al.[52]	https://bioconductor.org/packages/ChAMP
conumee2 R package	Daenekas et al.[53]	https://bioconductor.org/packages/conumee2
Rtsne R package	van der Maaten & Hinton[54]	https://cran.r-project.org/package=Rtsne
SeSAMe R package	Zhou et al.[55]	https://bioconductor.org/packages/sesame
InfiniumPurify R package	Zheng et al.[56]	https://cran.r-project.org/package=InfiniumPurify
mLiftOver	Chen & Zhou[57]	https://bioconductor.org/packages/mLiftOver
MethylCIBERSORT	Chakravarthy et al.[36]	https://cibersortx.stanford.edu/
QuPath	Bankhead et al.[58]	v0.6.0; https://qupath.github.io
Enrichr	Xie et al.[59]	https://maayanlab.cloud/Enrichr/
crossNN	Yuan et al.[38]	N/A
GraphPad Prism 10	Graphpad software	https://www.graphpad.com
Inkscape	Inkscape Project	https://inkscape.org/
SEQUENCE Pilot Software Version 5.4.0	JSI medical systems GmbH	https://www.jsi-medisys.de/

EXPERIMENTAL MODEL AND STUDY PARTICIPANT DETAILS

In this study, we included women patients from three different institutions: Charité, Universitatsmedizin Berlin (Berlin), Evangelische Kliniken Essen-Mitte (Essen), and Peter MacCallum Cancer Center (PMCC), Melbourne (Melbourne). In the Berlin cohort, we included all available MOC and EOM FFPE samples from the archive of the Institute of Pathology from 2000 to 2023, totaling 34 MOC and 37 EOM samples. In addition, we also included 18 mBOT samples for comparing MOC to its precursor lesions. For the Essen cohort, we included all MOC and EOM FFPE samples diagnosed between 2021 and 2023.

The PMCC collective comprised 18 MOC cases from the study by Cheasley et al., for which DNA was still available. These cases originated from the Australian Ovarian Cancer Study (AOCS; n = 4), the Edinburgh Cancer Research Centres (ECRC; n = 12), the Mayo Clinic (Mayo; n = 1), and the Victorian Cancer Biobank (VCB; n = 1). For all PMCC collective cases, genome-wide DNA methylation data, mutational profiles, patient age and survival information, and histopathological details including grade, T-stage, and IHC profiles were available. However, due to limited tissue availability in the PMCC cohort, *de novo* IHC staining could not be performed to complete the full IHC panel. As such, only IHC markers available from existing cohort metadata were included in the analysis. As part of inter-cohort IHC quality control, PanCK H-score distributions were compared between cohorts, and a cohort exhibiting significantly lower PanCK expression was excluded from subsequent analyses.

Normal ovary samples (*in-house*; n = 17) and normal Fallopian tube samples from University of Iowa (UIOWA; n = 12)[60] were included as healthy tissue controls.

For the Nanopore analysis, we included 14 FF MOC samples, 8 of which were matched to MOC FFPE cases of the main cohort, and 5 FF EOM samples (2 EOM^{STAD}, 1 EOM^{COAD}, 1 EOM^{PAAD}, 1 EOM$^{cervix\ adenocarcinoma}$). These samples were obtained from the Department of Gynecology in the Center for Oncological Surgery of the Charité-Universitätsmedizin Berlin.

For the external validation cohort, we included 21 additional MOC samples and 24 EOM samples from three different pathology institutions: Ludwig-Maximilians-Universität München (Munich), University Medical Center Hamburg-Eppendorf (Hamburg) and University Medical Center Göttingen, (Göttingen). All the samples of the external validation cohort underwent the same strict diagnosis review as the internal validation samples.

The study was approved by the ethics commissions of Charité-Universitätsmedizin Berlin (EA1/110/22).

METHOD DETAILS

Diagnosis review

To confirm the MOC diagnosis, each case file was clinicopathologically reassessed by the study's overseeing pathologist (MPD). Decisive criteria to rule out metastatic status were prior upper-GI gastroenterostomy and colonoscopy showing no evidence of malignancy, and, following the[14] evaluation method, which favors a primary ovarian origin when tumors are unilateral, exceed 10 cm in greatest dimension, and lack signet ring cell morphology. Immunohistochemical profiling was also incorporated into the diagnostic framework. The IHC panel included CK7, CK20, SMAD4, and PAX8, with additional confirmation given by low p16 expression and SMAD4 wild type pattern. The presence or absence and type of precursor lesions associated with MOC were obtained from the respective pathology reports and corroborated by histopathologic review of the tissue sections. For all EOM a primary tumor of origin was known or was detected during the immediate follow-up after the discovery of an ovarian tumor.

TMA construction

For all samples from the Berlin and Essen Cohort and for the samples of the external validation cohort for which material was available, we constructed a tissue microarray (TMA). Briefly, from tumor-rich areas, three 2 mm cores were either manually stamped out or automatically extracted using the TMA Grandmaster system (3DHISTECH, Budapest, Hungary). These cores were then embedded into recipient paraffin blocks. The blocks were categorized by tumor type, resulting in three MOC and three EOM blocks.

Immunohistochemistry staining and quantification, IHC classification

For immunohistochemical (IHC) analysis, 2 μm sections were prepared from the TMA blocks. Staining was performed using the BenchMark ULTRA system (Ventana Medical Systems, Tucson, AZ, USA) following the manufacturer's protocol. Antigen retrieval was carried out by incubating samples either at 95°C for 8 min in CC1 mild buffer or at 36°C in Protease 1, depending on the antibody. The sections were subsequently stained with the following antibodies: anti-ANXA1 (610067, Biosciences, 1:5000), anti-CDX2 (EPR2764YZ, Zytomed, 1:50), anti-CK7 (M7018, DAKO, 1:1000), anti-ER (790–4325, Ventana Roche, neat), anti-MUC5AC (18–2322, Leica, 1:50), anti-P16 (805–4713, Ventana Roche, neat), anti-p53 (M7001, DAKO, 1:50), anti-panCK (M0821, DAKO, 1:1000), anti-PAX8 (760–4618, Ventana Roche, neat), anti-PR (790–4296, Ventana Roche, neat), anti-SATB2 (ab92446, Abcam, 1:300), anti-SMAD4 (ab40759, Abcam, 1:200), and anti-CD8 (M7103, Dako, 1:100). Detection was performed using the UltraView Universal DAB Detection Kit, with hematoxylin used for counterstaining (12 min) and a bluing agent applied for an additional 12 min, all conducted on the BenchMark ULTRA system. Upon completion, slides were scanned using the PANNORAMIC 1000 (3DHISTECH) and Aperio GT 450 (Leica Biosystems) scanners.

The protein expression levels in the IHC-stained TMA slides were quantified using the histochemical score (H-score) system. This scoring method evaluates both staining intensity and distribution across tumor tissue.[61,62] Staining intensity is graded from 0 to 3: no

staining (L0), weak (L1), moderate (L2), and strong (L3). The percentage of tumor cells at each intensity level is calculated, and the H-score is determined using the following equation: H-score = $(0 \times L0) + (1 \times L1) + (2 \times L2) + (3 \times L3)$. The final H-score ranges from 0 to 300, representing the extent of staining across the tissue. Two pathologists independently estimated the percentage of positive cells in increments of 5%. The final H-score was calculated as the average of both assessments. For each tumor, the median H-score across the three TMA spots was calculated to derive the final score.

IHC results were used to classify samples as displaying either a MOC or EOM indicative or unspecific immunohistochemical profile, adapted from the AFIP[14] guidelines. A profile indicative of MOC was defined as SATB2-negative, CK7-positive, and PAX8-positive, irrespective of CK20 status. Conversely, EOM was favored in cases with SATB2-negativity and PAX8-negativity. Within this group, CK7-positivity supported an upper gastrointestinal or pancreatobiliary origin, while CK7-negativity combined with CK20-positivity suggested a lower gastrointestinal primary. SATB2-positivity strongly favored EOM. Regardless of PAX8 status, CK7-negativity and CK20-positivity supported appendiceal origin, while CK7-positivity and CK20-positivity was interpreted as consistent with colorectal metastasis.[22–25] $CD8^+$ cell proportions in MOC^{K1} and MOC^{K2} were quantified using QuPath 0.6.0.[58] Tumor and adjacent stroma areas were manually delineated on all available TMA spots of each sample, followed by automated positive cell detection. To assess the spatial distribution of $CD8^+$ T cells, a tumor-to-stroma infiltration index was calculated as Infiltration = log2 x [(tumor% + 0.01)/stroma% + 0.01)], consistent with prior spatial immunophenotyping approaches that use log2 ratios to assess immune cell localization.[63] To permit calculation for zero values, a pseudocount of 0.01 was added.

For the external validation cohort, we performed the IHC staining necessary for IHC classification: CK7, CK20, SATB2 and PAX8.

DNA extraction

For all MOC and EOM samples, tumor-rich areas were marked, and the tumor cell content was determined using a light microscope (Olympus, BX46) by one of the study pathologists (MPD). For DNA extraction, punch biopsies of the FFPE block were performed from regions showing unequivocal invasive carcinoma morphology and high histological grade, and independently confirmed by a second pathologist prior to tissue sampling. For mBOT samples, because of their cystic nature, the entire tumor contour on one slide was marked and up to 20, 5 µm thick slides were cut and the surface was scratched for DNA extraction. Normal ovary samples were obtained from patients undergoing surgery for benign ovarian/uterine lesions, with tissue collected for DNA methylation from areas at the interface of the surface epithelium and stroma, always containing surface epithelium. We performed semi-automated DNA extraction according to the manufacturer's instructions (Maxwell RSC FFPE Plus DNA Purification Kit, Custom, Promega). DNA quantities were measured using Qubit HS DNA assay (Thermo Fisher Scientific).

DNA methylation

As input, we used 500 ng of DNA for the DNA methylation analysis. The Illumina Infinium HD FFPE DNA Restore Kit (Illumina, CA, USA) was used for DNA restoration from FFPE samples. Next, we used the EpiTect Bisulfite Kit (Qiagen) for bisulfite conversion. The arrays were used according to the manufacturer's instructions for the DNA methylation analysis of all MOC and EOM samples. For mBOT, 10 samples were analyzed with the EPICv1 array, while the other 8 were analyzed with the EPICv2 array, as the EPICv1 was not produced by the manufacturer anymore. All 45 samples of the external validation cohort were analyzed using the EPICv2 array.

DNA panel sequencing and mutation call

The Ion AmpliSeq Library Kit 2.0 (Thermo Fisher Scientific, Waltham, MA, USA) was used to perform library preparation of 80 ng of genomic DNA using the Ion AmpliSeq Cancer Hotspot Panel v2 (Thermo Fisher Scientific), covering 50 oncogenes and tumor suppressor genes. Genes and the respective exons covered by the NGS panel are listed in the supplementary material (Table S1). The final library was quantified using the Ion Library Quantitation Kit (Thermo Fisher Scientific). Samples were multiplexed and amplified on Ion Sphere Particles with Ion 540 Kit-Chef and were sequenced using Ion 540 Chips on an ION S5 Instrument (Thermo Fisher Scientific).

Variants with an allele frequency greater than 5% were selected. Mutation calling was conducted using SEQUENCE Pilot Software, Version 5.4.0 (JSI medical systems GmbH, Ettenheim, Germany), aligned by using the human genome (hg19) as reference, and only variants considered pathogenic and potentially pathogenic were selected for further analysis. Samples in which sequencing failed or yielded insufficient data quality were excluded from the mutational analysis.

DNA methylation array processing

The raw data in IDAT files was preprocessed in R using various packages as implemented in ChAMP.[52] Each array type was preprocessed separately. The minfi package[64] was used to load the methylation signals, followed by quality control, which removed CpG sites with a detection p-value above 0.01 and low-quality sites, with less than 3 beads in at least 5% of the samples. Furthermore, all SNP-related sites,[52] multi-hit sites,[52] and CpGs located on the X and Y chromosomes were removed. The resulting beta values were normalized using FunNorm.[65] Lastly, the EPICv1 and EPICv2 datasets were merged by keeping only the common CpGs between the two arrays.

Tumor purity estimation

To estimate tumor purity, EPICv1 and v2 samples were first converted to 450k ,[57] and purity was then inferred with the InfiniumPurify R package[56] using normal ovary samples as reference.

t-distributed stochastic neighbor embedding (t-SNE) and cluster analysis

t-SNE dimensionality reduction was performed on the preprocessed DNA methylation beta values using the Rtsne package[54] in R. The samples for each tissue type were analyzed based on the 20,000 most variable CpG sites, determined by standard deviation. Prior to t-SNE embedding, the Rtsne implementation performs an initial principal component analysis (PCA) to reduce dimensionality. The retained principal components explained 44.31% and 16.06% of the variance for the MOC and mBOT plots, and 42.0% and 13.4% of the variance for the MOC, mBOT, and EOM plots. The t-SNE algorithm was executed with 5,000 iterations and a perplexity of 9 for the MOC and mBOT plots, a perplexity of 7 for the MOC, mBOT and EOM plots, and a perplexity of 30 for the multi-tumor landscape.

Copy number analysis

Copy number variation (CNV) analysis was performed using the conumee2 package[53] in R. Illumina EPICv1 and EPICv2 methylation raw IDAT files were processed with SeSAMe[55] to extract probe intensities. EPICv2 data were harmonized to EPICv1 using mLiftOver.[57] Normal ovary samples (n = 17) were used as controls for CNV fitting. CNV summary plots were generated for: $mBOT^{normal\_like}$ (n = 8), $mBOT^{MOC\_like}$ (n = 10), MOC^{K1} (n = 38), MOC^{K2} (n = 20), all MOC samples combined (n = 58) and EOM (n = 38). Gains and losses were called using a log2 ratio threshold of ±0.1. Genes were annotated using the UCSC hg19 genome, and selected genes of interest were highlighted on the CNV plots.

Differently methylated CpG probes and pathway analysis

Differential methylation analysis was performed on the pre-processed beta values using the ChAMP R package.[52] After imputation, pairwise comparisons between sample groups were conducted, selecting CpGs with an adj. $p < 0.01$ and an absolute logFC $\geq$0.3. The hypermethylated and hypomethylated CpGs were matched to their corresponding gene location. For pathway analysis, only genes with differential methylation at the transcription start site (TSS) were selected. These TSS-specific gene lists from hyper- and hypomethylated CpGs were analyzed separately using the Enrichr online platform with the Reactome 2024 pathway database to identify the top enriched pathways.[59,66,67]

Clustering analysis

K-means clustering was performed in R on the top 20,000 most variable CpG sites from the preprocessed data, selected based on standard deviation across MOC samples. Beta values were scaled prior to clustering. The optimal number of clusters was determined using the elbow method based on the within-cluster sum of squares,[68] and clusters were visualized by PCA. PCA was performed for visualization, and the first two principal components explained 13.37% and 6.04% of the total variance, respectively. The analysis was implemented using the matrixStats[69] and ggplot2[70] packages. Hierarchical clustering was performed using Ward's method on Euclidean distance, and methylation heatmaps with overlaid dendrograms were generated using pheatmap.[71] To validate cluster assignments in the external validation cohort, cluster centroids were defined as the mean scaled beta value across the top 20,000 variable CpGs for all samples within each cluster (K1 and K2) of the internal cohort. Each external validation sample was assigned to the nearest centroid based on Pearson correlation, with the assignment determined by the higher correlation coefficient between the sample's scaled methylation profile and each centroid.

Cell type deconvolution

Cell type proportions of the methylation samples were estimated using the MethylCIBERSORT tool.[36] An ovarian cancer-specific signature matrix generated by Biskup et al.[35] was used as the signature input file and a separate mixture matrix was created from the preprocessed beta values (see previous step for preprocessing details) per cancer type. The files were uploaded onto https://cibersortx.stanford.edu/ and the cell fraction deconvolution was subsequently performed at 500 permutations.

Other DNA methylation sets used for training and testing the classifier

In addition to the samples already described in this study, we incorporated external DNA methylation datasets generated on EPICv1 and EPICv2. These datasets included both FFPE and fresh frozen (FF) material and were used for classifier training and testing.

Step 1 training: "Other" class samples: 17 breast carcinoma (BRCA; FFPE and FF, GEO: GSE212370),[72] 20 colorectal adenocarcinoma (COAD; FFPE, GEO: GSE233854),[73] 17 high-grade serous ovarian carcinoma (HGSOC; FFPE, *in-house*), 16 intrahepatic cholangiocarcinoma (iCCA; FFPE, *in-house*), 14 low-grade appendiceal mucinous neoplasms (LAMN; FFPE, *in-house*), 4 lung adenocarcinoma (LUAD; FFPE, *in-house*), 6 LUAD (FF, GEO: GSE249157),[74] 20 pancreatic adenocarcinoma (PAAD; FFPE, *in-house*), 20 stomach adenocarcinoma (STAD; FFPE, *in-house*), 16 uterine corpus endometrial carcinoma (UCEC; FF, GEO: GSE178610),[75] 12 normal Fallopian tube samples (FF, GEO: GSE133556),[60] and 10 normal ovary samples (FFPE, *in-house*).

Step 2 training: The "primary Mucinous Carcinoma" class was built from the same datasets as in step 1 (17 BRCA, 20 COAD, 16 iCCA, 14 LAMN, 10 LUAD, 20 PAAD, 20 STAD, and 16 UCEC).

The "no match" controls included overlapping datasets from step 1 (17 HGSOC, 12 normal Fallopian tube, 10 normal ovary) plus additional cohorts: 16 hepatocellular carcinoma (HCC; FF, GEO: GSE136583),[76] 18 head and neck squamous cell carcinoma (HNSC; FFPE, GEO: GSE124052),[19] 4 lung squamous cell carcinoma (LUSC; FF, GEO: GSE249157),[74] 10 LUSC (FFPE, GEO: GSE124052),[19]

CellPress
OPEN ACCESS

5 pancreatic neuroendocrine carcinoma (Pan-NEC; FFPE, Benhamida et al.),[77] 18 pancreatic neuroendocrine tumor (Pan-NET; FFPE, Benhamida et al.),[77] and 6 pancreatic solid pseudopapillary neoplasms (Pan-SPN; FFPE, Benhamida et al.).[77]

Step 3 training: STAD class: 20 STAD (FFPE, *in-house*; also included in step 1). COAD class: 20 COAD (FFPE, GEO: GSE233854),[73] also included in step 1). "Other EOM" class: using the same from step 1 (17 BRCA, 16 iCCA, 14 LAMN, 10 LUAD, 20 PAAD, and 16 UCEC).

The test cohort was divided into three groups: Primary mucinous carcinoma (pMC) class: 20 BRCA (FFPE and FF, GEO: GSE212370),[72] 9 COAD (FFPE, *in-house*), 11 COAD (FFPE, GEO: GSE233854),[73] 16 iCCA (FFPE, *in-house*), 4 LAMN (FFPE, *in-house*), 3 LUAD (FFPE, *in-house*), 4 LUAD (FF, GEO: GSE249157),[74] 16 PAAD (FFPE, *in-house*), 12 STAD (FFPE, *in-house*), and 20 UCEC (FF, GEO: GSE178610).[75] Other lesions class: 11 HCC (FF, GEO: GSE136583),[76] 18 HNSC (FFPE, GEO: GSE124052),[19] 15 LUSC (FFPE, GEO: GSE124052),[19] 5 Pan-NEC (FFPE, Benhamida et al.),[77] 20 Pan-NET (FFPE, Benhamida et al.),[77] 6 Pan-SPN (FFPE, Benhamida et al.),[77] and 20 HGSOC (FF, GEO: GSE133556).[60] Normal ovary class: 7 normal ovary samples (FFPE, *in-house*).

Classification pipeline development

The classification pipeline consists of three successive steps. Steps one and two each consist of a binomial classifier; step three comprises two binomial classifiers. Each step was trained independently. Every binomial classifier focuses on identifying a particular class, with the negative class treated as "other." If, at any step, the positive class is assigned, that class becomes the final outcome and subsequent steps are not executed.

Step one distinguishes MOT from other tumor types; if positive, MOT is the final class. Samples classified as "other" in step one proceed to step two, which determines whether the sample is MC or another tumor type. A sample labeled "other" in both steps one and two is assigned the class no-match. Samples classified as MC in step two proceed to step three: the first classifier evaluates whether the MC has a COAD origin (if positive, MC-COAD is assigned); if negative, the second classifier evaluates whether the MC has a STAD origin (if positive, MC-STAD is assigned). If neither classifier in step three returns a positive result, the sample is assigned MC-unknown.

The same feature-selection process was conducted separately for each model on its own training set. First, the iDAT files were loaded using the Python library mepylome[78]; beta values extracted and normalized using noob.[64] In order to preserve compatibility between EPICv1 and EPICv2 arrays, only the common CpGs between the two arrays were selected. Furthermore, because the positive class samples in the step 3 STAD classifier were scanned using the EPICv2 array while the negative class samples were scanned with the EPICv1 array, there was a high risk of the model learning the differences between arrays instead. To account for that, the CpG set for step 3 was restricted to the array invariant probes and adjusted the biases according to the values in mLiftOver.[57] The same bias correction was applied during prediction of EPICv2 samples. From the resulting set the top 5,000 CpGs by standard deviation were selected; pairwise correlations were then computed, and for any pair with an absolute correlation >0.9, the CpG with the lower standard deviation was removed. The remaining CpGs were transformed using PCA, and the resulting components were used as features. The number of components was one of the hyperparameters optimized.

For each binomial classifier, we trained two machine learning models in Python using the library sklearn,[79] a logistic regression (LR) and a support vector machine (SVM), evaluated them independently, and retained the better model. We used the Python library optuna[80] with 5-fold cross-validation for hyperparameter tuning. Due to the class imbalance in every training set, we decided to use the G-mean, defined as the square root of sensitivity times specificity, as the optimization goal and main evaluation criterion for our models. The same search procedure and space were used for every step, but tuning was conducted independently per classifier. For LR, we optimized the solver (liblinear, saga, lbfgs), penalty (l1, l2, none), inverse regularization strength C (0.0001–10), and class_weight (balanced, none). For SVM, we optimized the kernel (search space: linear, rbf, poly, or sigmoid), the regularization parameter "C" (search space between: 0.001 and 100), and class imbalance "class_weight" (search space: balanced or none). Additionally, we optimized for the "poly" kernel only, the degree (2, 3, 4, 5), and for kernels "rbf", "poly", "sigmoid", the gamma type (scale, auto, or float). For the gamma type "float", the gamma value (0.0001–1) was also optimized.

DNA methylation intratumoral heterogeneity

To assess the impact of morphological heterogeneity on subclassification into K1 and K2 and on the diagnosis of MOT versus EOM, DNA methylation profiling was performed on two spatially distinct tumor regions from three MOC samples of the external validation cohort. DNA was obtained from FFPE tissue sections using microdissection to isolate the selected regions. For the first sample, areas corresponding to mBOT and MOC G1 morphology were selected. For the second sample, one region with G1 morphology and another with G2 morphology were analyzed. For the third sample, one region exhibiting expansile growth and another showing infiltrative morphology were profiled. The resulting methylation profiles were then compared to evaluate the consistency of classification and subclassification between the spatially distinct tumor regions. For the primary analysis the more aggressive morphology was used: MOC, G2, and infiltrative, respectively.

Nanopore low-pass whole genome sequencing

200 ng genomic DNA were used for transposase-based library preparation (Rapid Barcoding Kit V14, Oxford Nanopore Technologies) according to the manufacturer's instructions. Multiplex libraries of four to five samples were sequenced on an R10.4.1 flow cell

(Oxford Nanopore Technologies) for 24 hours on GridION device (Oxford Nanopore Technologies). Raw data were basecalled using high-accuracy (HAC) dorado models with modified basecalling of 5mC and 5hmC in CpG contexts. Unaligned BAM files were processed using nanoDx, version 1.2.[81] Briefly, reads were aligned to the hg19 reference genome using minimap2[82] v.2.26 and CpG methylation calls were aggregated using modkit v.0.2.3.

Methylation-based classification from sparse methylomes

For DNA methylation-based classification of Nanopore sequencing data, a crossNN neural network model was trained as described previously.[38] The training set comprised microarray-based methylation data (beta values) from a previously described pan-cancer reference set,[38] complemented with the MOC reference cases (n = 20; the same ones from the main classifier) and normal ovary controls (n = 17) described above. The model was trained with a masking rate of 99.5% for 3,000 epochs. To determine optimal cut-offs for the classification score, ROC analysis was performed and internally cross-validated in n = 5379 independent cases profiled on different platforms (randomly sampling 50% of the validation cohort). For each fold and methylation profiling platform, the cut-off at the Youden index was determined. A final cut-off of >0.2 and >0.6 was manually selected for predictions from Nanopore sequencing and microarray data, respectively. Predictions were made using crossNN models as implemented in nanoDx. For classification of microarray cases using crossNN, IDAT files were preprocessed using minfi[64] using the illuminaPreProcess method and beta values were converted to bedMethyl-style format before being used as input to crossNN. Cases with classification scores below cut-offs were considered "not classifiable".

QUANTIFICATION AND STATISTICAL ANALYSIS

All statistical analyses and graph generation were performed using GraphPad Prism 10. For comparisons between groups, normality was first assessed. Depending on the data distribution, either an unpaired two-tailed *t* test (for normally distributed data) or the Mann-Whitney U test (for non-normally distributed data) was applied. Categorical variables were compared using Fisher exact test. Comparisons of estimated cell-type proportions across groups were performed using the one-way ANOVA or the Kruskal–Wallis test, selected based on normality testing (Shapiro–Wilk), reported pairwise p-values derive from post hoc multiple-comparison testing: Tukey's test following ANOVA and Dunn's test following Kruskal-Wallis.

Overall survival was defined as the time from the date of diagnosis to death from any cause or last follow-up. Patients alive at last follow-up were censored. Survival analyses in Figure 2 were conducted using the Mantel–Cox log rank test. The maximum follow-up time was truncated at 10 years, and the median follow-up duration for the cohort was 9.1 years. A multivariate Cox proportional hazards regression model in Figure 2 was employed to assess the independent association of parameters that demonstrated significance in univariate survival analyses using the Mantel-Cox log rank test. Potential confounding between covariates was evaluated using Fisher's exact test, and multicollinearity was assessed via variance inflation factors. The proportional hazards assumption was tested for each covariate and globally using Schoenfeld residuals and associated statistical tests. Model fit was assessed using the likelihood ratio, Wald, and score (log rank) tests. Discriminative ability was evaluated using Harrell's concordance index (C-index). For all statistical tests a two-sided p-value <0.05 considered statistically significant.

Cell Reports Medicine, Volume 7

Supplemental information

DNA methylation profiling enables subclassification of mucinous ovarian carcinoma and distinguishes it from extraovarian mucinous metastases

Teodor G. Calina, Elena Grafenhorst, Simon Schallenberg, Markus Möbs, Bjarne Daenekas, Alexandru A. Sabo, BaoQing Chen, Ines Koch, Tobias Janik, Michael Churchman, Charlie Gourley, Elizabeth L. Christie, Catalin Vasilescu, Vlad Herlea, Wolfgang D. Schmitt, Nicolae Leopold, Florian Heitz, Angelina Vlaski, Yuri Tolkach, Felix Bremmer, Frederick Klauschen, Reinhard Büttner, Philipp Ströbel, Eike Burandt, Guido Sauter, Frank L. Heppner, Eliane T. Taube, Jalid Sehouli, Ioana E. Braicu, Philipp Euskirchen, David Horst, Kylie L. Gorringe, David Capper, and Mihnea P. Dragomir

Supplementary Figures

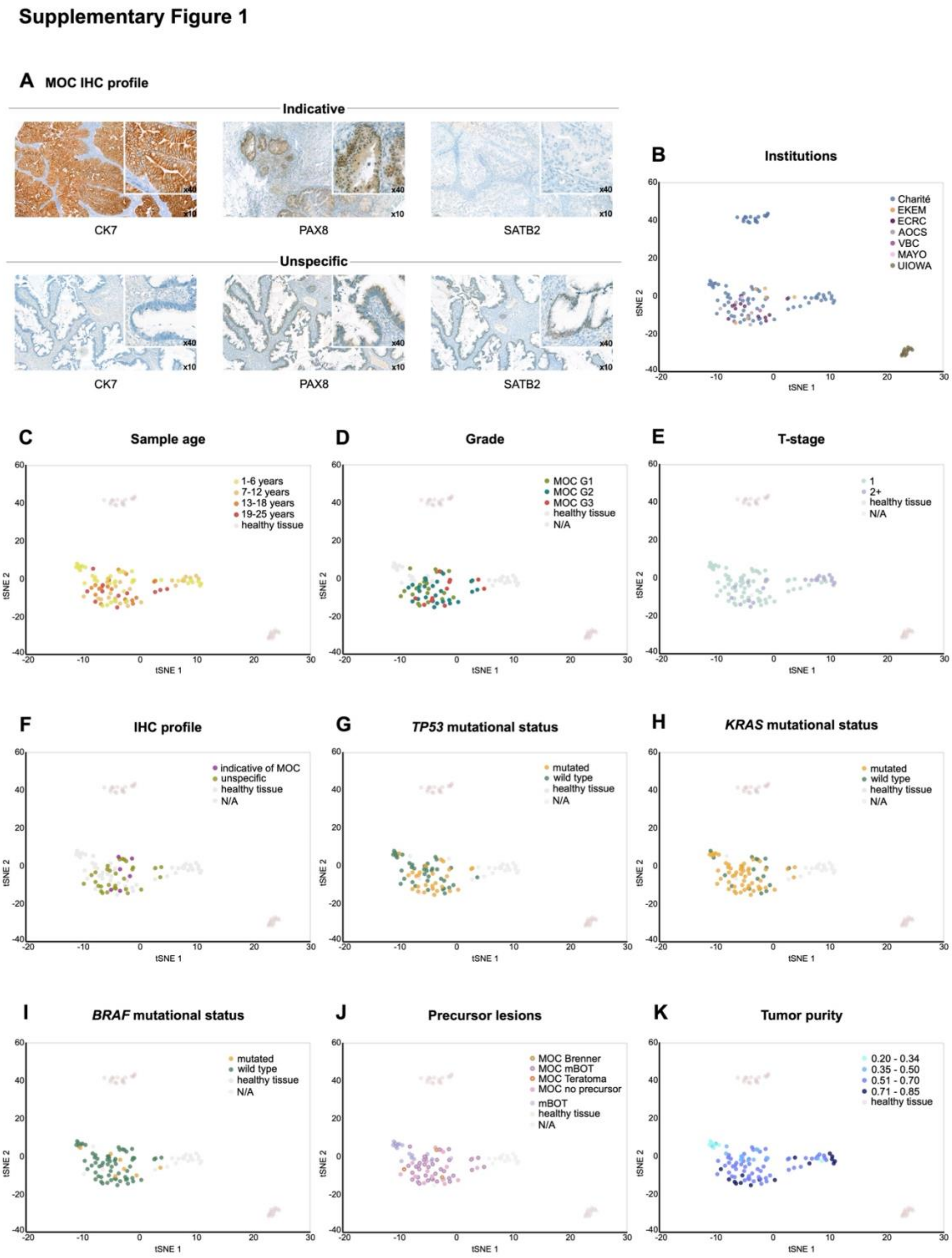

Figure S1. Characterization of the mucinous ovarian carcinoma (MOC) cohort. Related to Figures 1.
(A) Representative IHC images of CK7, PAX8, and SATB2 staining in MOC with indicative (upper panel) or unspecific IHC profiles (lower panel).
(B–K) t-SNE plots of MOC and other ovarian lesions and normal tissues colored by (B) institution, (C) sample age, (D) grade, (E) T-stage, (F) IHC profile, (G) *TP53* mutational status, (H) *KRAS* mutational status, (I) *BRAF*

mutational status, (J) precursor lesions (Brenner tumor, mBOT, teratoma, no precursor, and N/A), and (K) tumor purity.

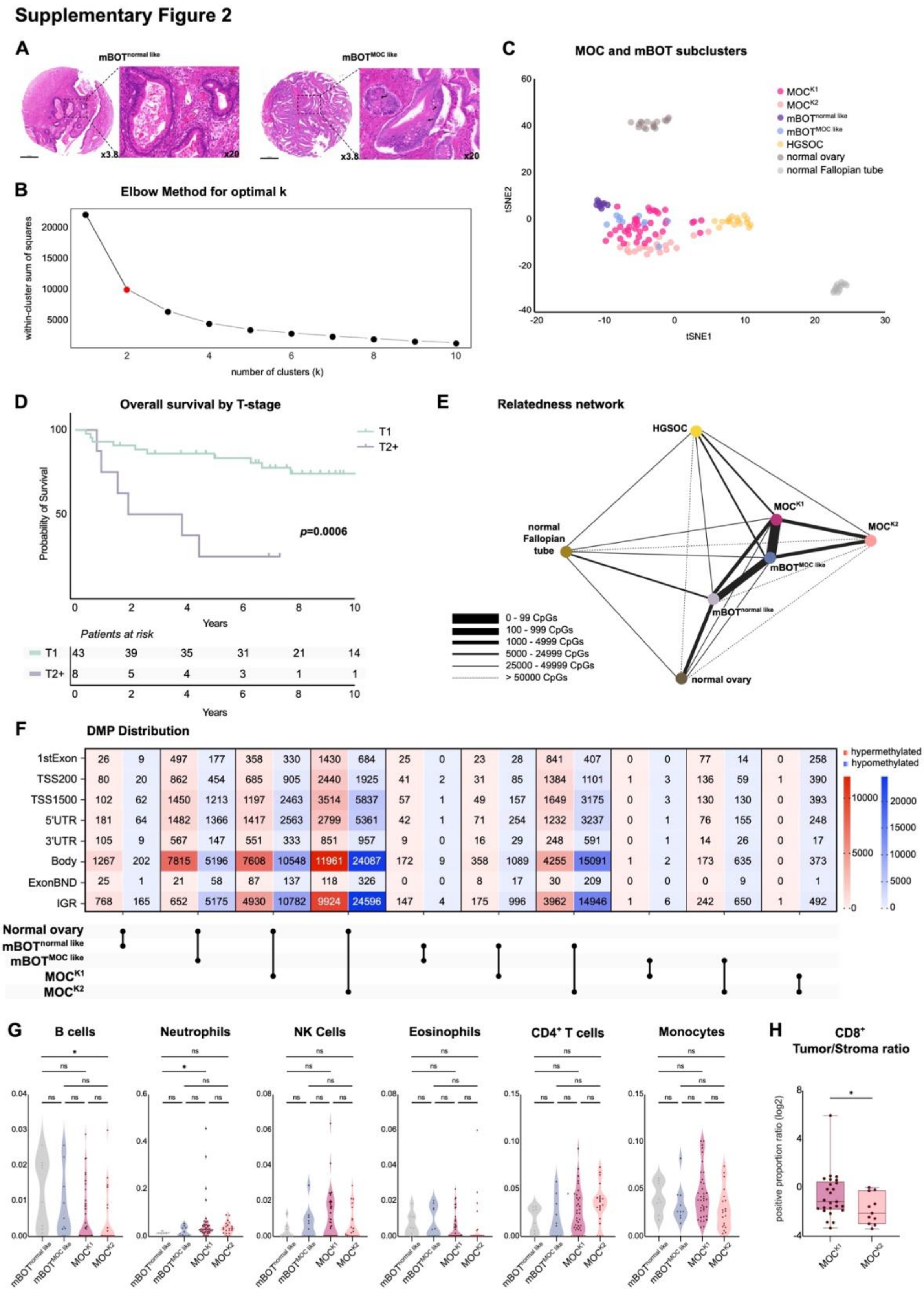

Figure S2. Characterization of mBOT and MOC subtypes. Related to Figures 2.

(A) Representative H&E images of mBOT subtypes at low (×3.8) and high (×20) magnification. Arrows marking mitoses. Scale bars, 500 µm.

(B) Elbow plot of within-cluster sum of squares used to determine the optimal k-means cluster number.

(C) t-SNE plot (same as in Fig. 1D) highlighting $mBOT^{normal\_like}$, $mBOT^{MOC\_like}$, MOC^{K1}, and MOC^{K2} subclusters, along with normal ovary, Fallopian tube, and HGSOC.
(D) Kaplan–Meier survival of MOC patients stratified by T-stage. *P* value from Mantel–Cox log-rank test.
(E) Relatedness network of methylation subtypes showing connections among $mBOT^{normal\_like}$, $mBOT^{MOC\_like}$, MOC^{K1}, MOC^{K2}, normal ovary, normal Fallopian tube, and HGSOC. Node size reflects group size, and edge thickness indicates the number of differentially methylated CpGs.
(F) Heatmap of differentially methylated probes (DMPs) across genomic features. Color intensity represents frequency of hypermethylated (red) and hypomethylated (blue) probes.
(G) Estimated immune cell proportions (y axis, % of positive cells) for B cells, neutrophils, NK cells, eosinophils, $CD4^+$ T cells, and monocytes in mBOT and MOC subtypes. Comparisons by Kruskal–Wallis with Dunn's correction. ns, not significant; $*p < 0.05$.
(H) Tumor-to-stroma ratio of $CD8^+$ T cell positive proportion in MOC^{K1} and MOC^{K2}. Data are shown as log2-transformed ratios; statistical comparisons were made using Mann Whitney test with $*p < 0.05$. Positive values indicate relative enrichment of $CD8^+$ T cells within tumor regions, whereas negative values indicate relative enrichment within the stromal compartment.

Supplementary Figure 3

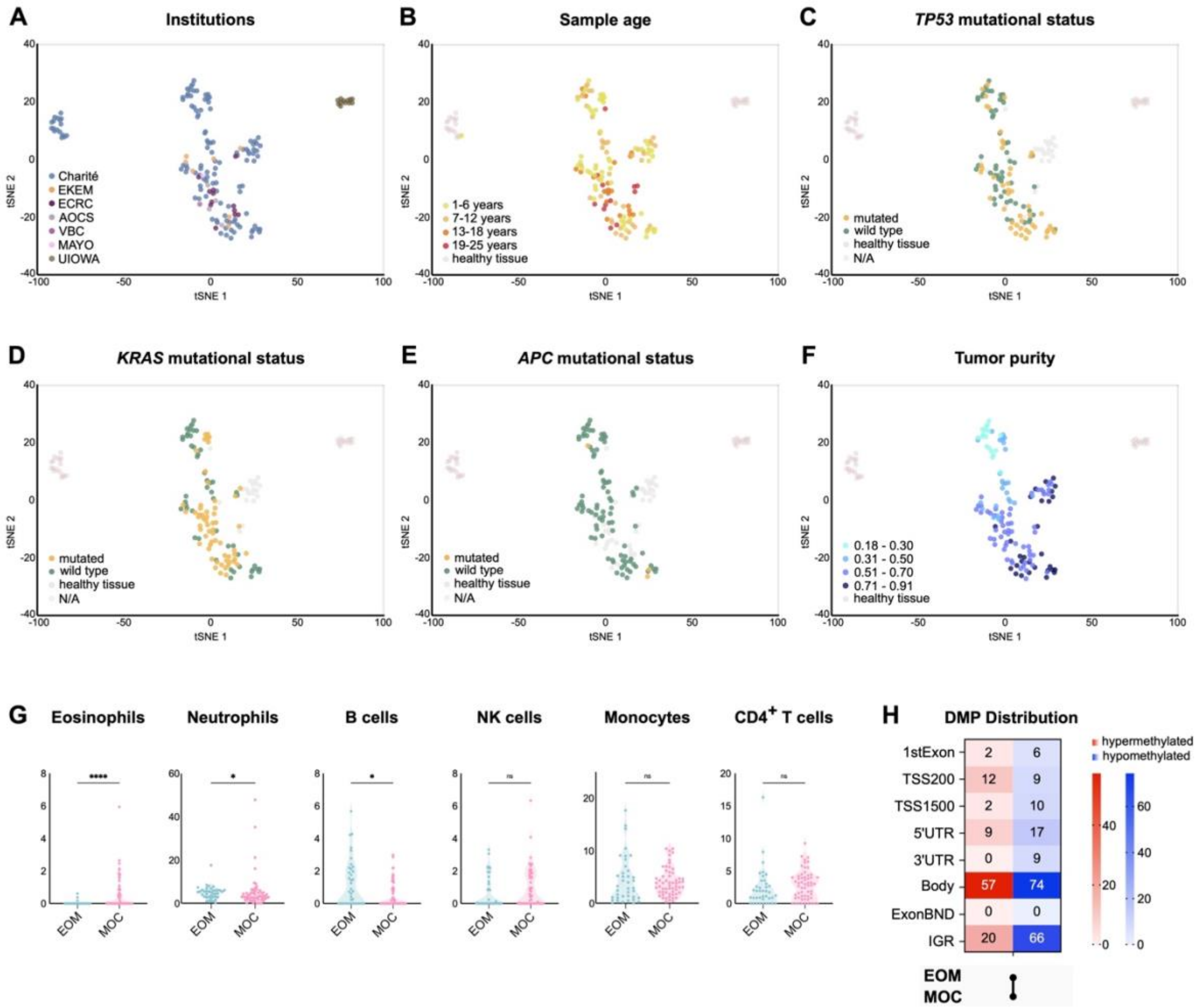

Figure S3. Characterization of the extra-ovarian metastasis (EOM) cohort. Related to Figures 3.
(A–F) t-SNE plots of MOC and related tissues colored by (A) institution, (B) sample age, (C) *TP53* mutational status, (D) *KRAS* mutational status, (E) *APC* mutational status, and (F) tumor purity.
(G) Estimated immune cell proportions (y axis, % of positive cells) for eosinophils, neutrophils, B cells, NK cells, monocytes, and $CD4^+$ T cells in EOM and MOC. Comparisons by Kruskal–Wallis with Dunn's correction. ns, not significant; *$p < 0.05$; ****$p < 0.0001$
(H) Heatmap of DMP distribution across genomic features comparing EOM and MOC. Color intensity represents frequency of hypermethylated (red) and hypomethylated (blue) probes.

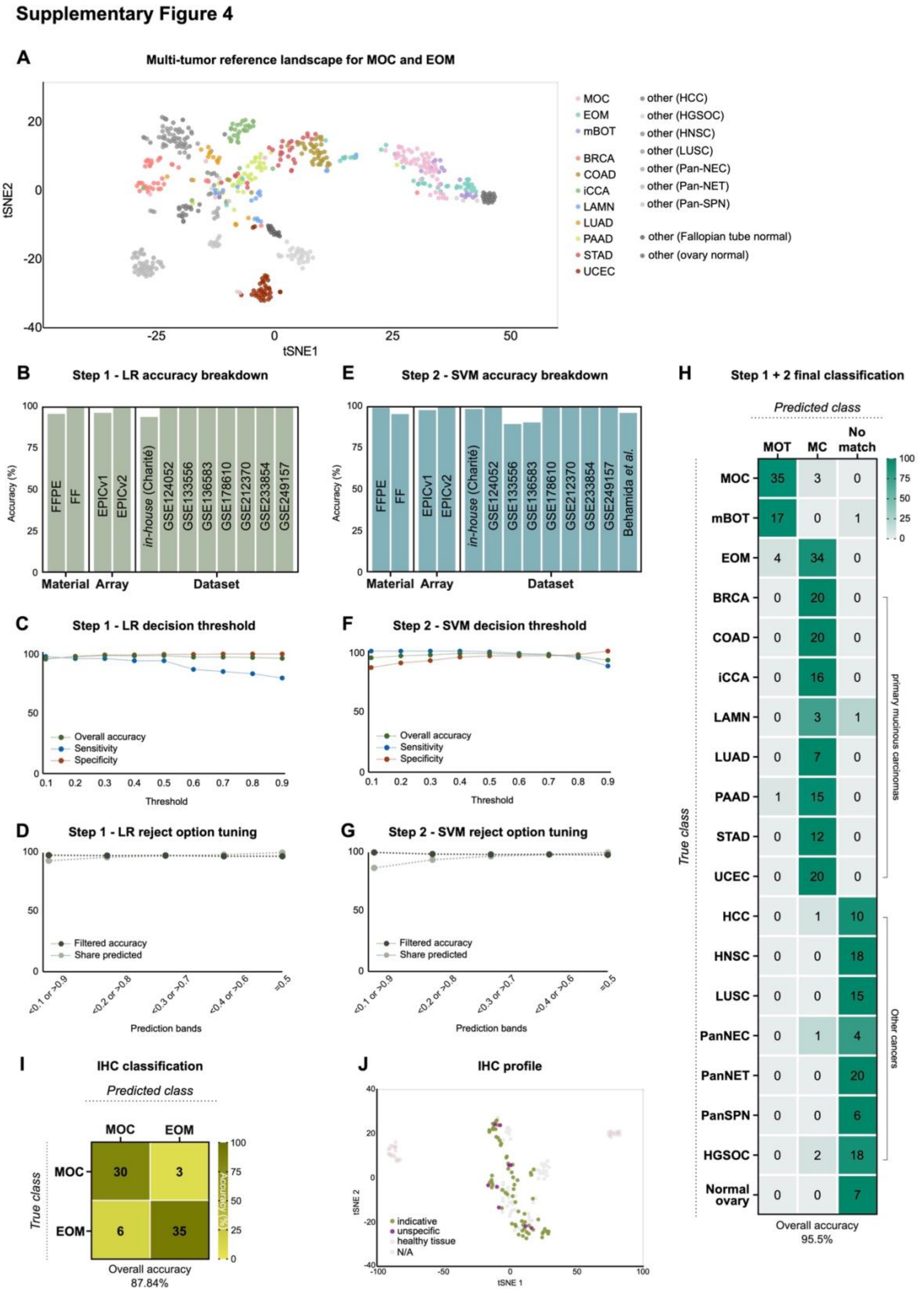

	MOT	MC	No match
MOC	35	3	0
mBOT	17	0	1
EOM	4	34	0
BRCA	0	20	0
COAD	0	20	0
iCCA	0	16	0
LAMN	0	3	1
LUAD	0	7	0
PAAD	1	15	0
STAD	0	12	0
UCEC	0	20	0
HCC	0	1	10
HNSC	0	0	18
LUSC	0	0	15
PanNEC	0	1	4
PanNET	0	0	20
PanSPN	0	0	6
HGSOC	0	2	18
Normal ovary	0	0	7

	MOC	EOM
MOC	30	3
EOM	6	35

Figure S4. Performance of the step 1 and step 2 classifiers and of the IHC classifier. Related to Figures 4. (A) t-SNE visualization of the multi-tissue landscape used for classifier development and internal validation. (BRCA: breast adenocarcinoma; COAD: colorectal adenocarcinoma; iCCA: intrahepatic cholangiocarcinoma; LAMN: low-grade appendiceal mucinous neoplasm; LUAD: lung adenocarcinoma; PAAD: pancreatic

adenocarcinoma; STAD: stomach adenocarcinoma; UCEC: uterine corpus endometrial carcinoma; HCC: hepatocellular carcinoma; HGSOC: high-grade serous ovarian cancer; HNSC: head and neck squamous cell carcinoma; LUSC: lung squamous cell carcinoma; Pan-NEC: pancreatic neuroendocrine carcinoma; Pan-NET: pancreatic neuroendocrine tumor; Pan-SPN: pancreatic solid pseudopapillary neoplasms).
(B–D) Step 1 logistic regression (LR) classification: (B) accuracy across material type, array platform, and dataset. (*In-house (Charité)* refers to all samples available in-house, including those from the Charité, EKEM, and the PMCC collective); (C) performance across decision thresholds (0.1–0.9) for overall accuracy, sensitivity, and specificity; (D) reject-option analysis showing filtered accuracy and predicted share.
(E–G) Step 2 support vector machine (SVM) classification: (E) accuracy across material type, array platform, and dataset. (*In-house (Charité)* refers to all samples available in-house, including those from the Charité, EKEM, and the PMCC collective); (F) performance across thresholds; (G) reject-option analysis.
(H) Confusion matrix for combined two-step classification (Step 1 LR + Step 2 SVM) across all classes (overall accuracy: 95.5%).
(I) Confusion matrix for IHC-based classification of MOC versus EOM (overall accuracy: 87.8%).
(J) t-SNE plot (same as in Fig. 3C) showing samples classified as indicative versus unspecific MOC based on IHC profile.

Supplementary Figure 5

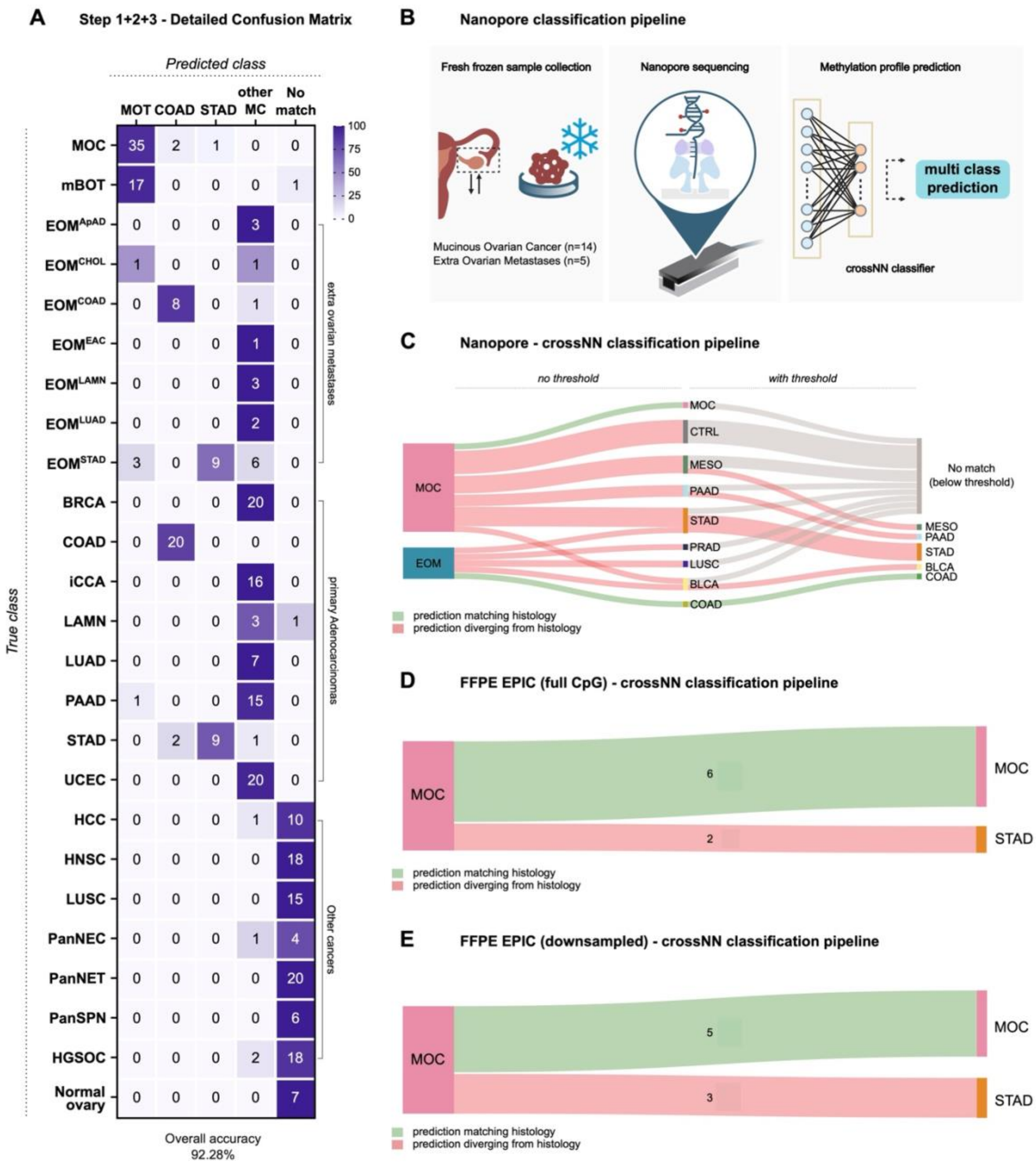

Figure S5. **Expanded classifier performance (Step 1+2+3), Nanopore diagnostic pipeline and results with and without CpG downsampling. Related to Figures 5.**

(A) Confusion matrix summarizing the final three-step DNA methylation classifier (Steps 1-3) across 25 histological classes/anatomical sites (overall accuracy: 92.28%).

(B) Schematic of the Nanopore-based classification workflow: fresh frozen samples undergo Nanopore sequencing, followed by methylation profile prediction and classification into MOT or EOM using the crossNN model. We deployed an expanded crossNN classifier version (trained on 20 MOC samples). Created with BioRender.com.

(C) Sankey plot showing per-sample Nanopore classification results using the crossNN classifier. Predictions are compared with histopathological diagnosis; green lines indicate concordance, and red lines indicate divergence. The classifier was tested on 19 intraoperative FF samples (14 MOC, 5 EOM). Results show that only 1/14 MOC

samples was correctly identified via Nanopore sequencing, with the majority of cases misclassified as non-MOC tumor types or healthy control tissue. Notably, one MOC sample containing Brenner tumor components was misclassified as bladder cancer (BLCA), likely due to shared urothelial differentiation signatures.

(D) Comparative Analysis of Matched EPIC Array and Nanopore Cohorts. To isolate the cause of low Nanopore accuracy, we analyzed a subset of 8 MOC cases profiled by both EPIC array (FFPE) and Nanopore (FF). When utilizing the full CpG set of the EPIC array, 6/8 samples were correctly classified as MOC. This indicates that the methylation signatures required for identification are present in the tumor tissue, though secondary misclassifications as stomach adenocarcinoma (STAD) occurred in two instances.

(E) Impact of CpG Coverage and Sampling on Diagnostic Sensitivity. To determine if Nanopore's low accuracy was a result of reduced sequencing depth, we downsampled the EPIC array data to match the Nanopore coverage (~3.61% of total CpGs). Despite this significant data reduction, 5/8 FFPE samples remained correctly classified. This markedly outperforms the 1/8 accuracy achieved using true Nanopore FF tissue sequencing on the same cases. These findings suggest that the diagnostic discrepancy is driven less by sequencing depth and more by intraoperative sampling factors, such as low tumor purity in FF samples compared to the curated FFPE blocks used for EPIC profiling.

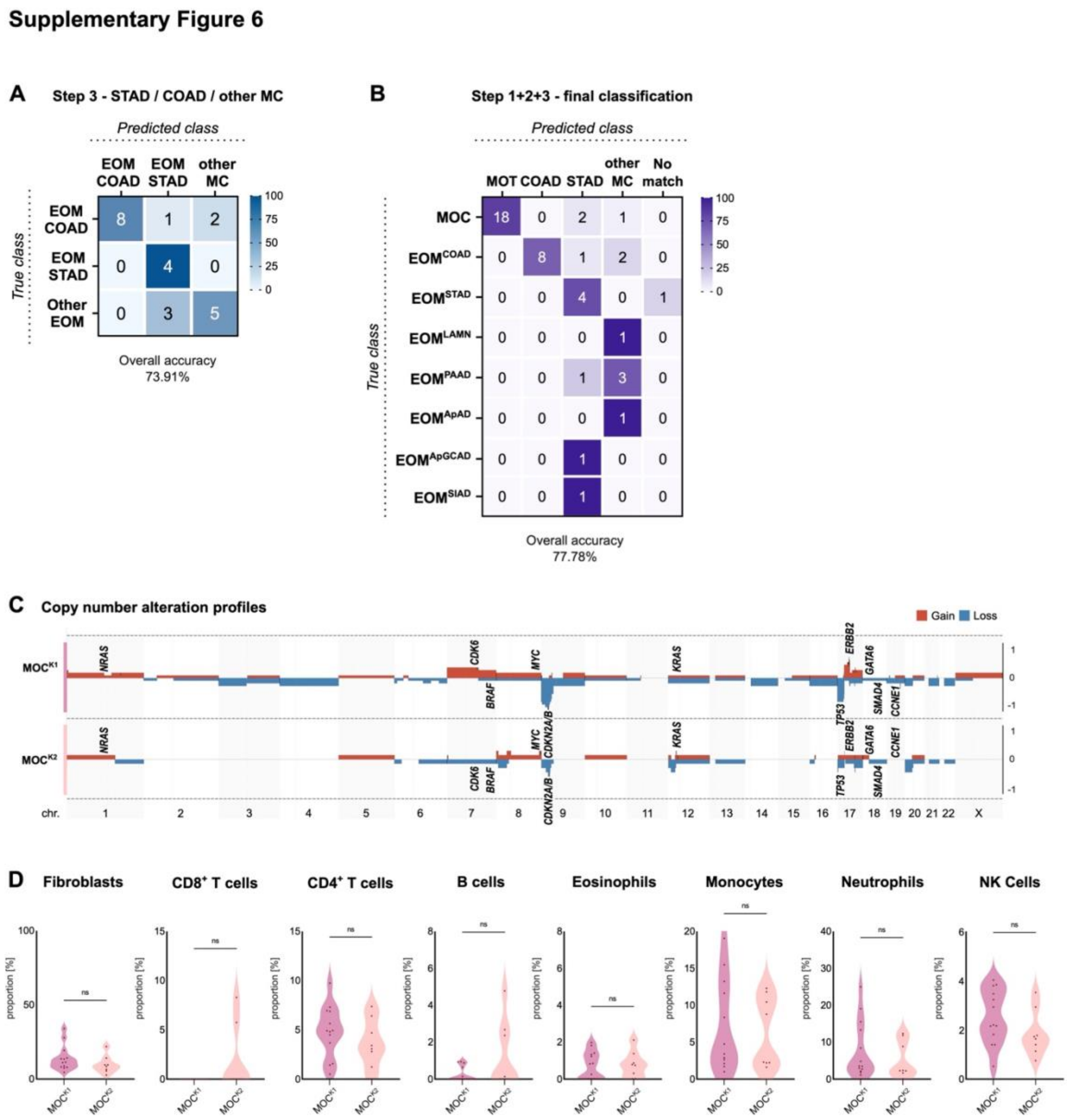

Figure S6. External validation of the classifier and DNA methylation subtypes of MOC. Related to Figures 6.

(A) Confusion matrix showing Step 3 classification results in the external validation cohort.

(B) Confusion matrix summarizing the final three-step DNA methylation classifier (Steps 1-3) in the external validation cohort.

(C) Genome-wide copy number alteration profiles for MOC^{K1} (external validation, n = 13), and MOC^{K2} (external validation, n = 8). The frequency of chromosomal gains (red) and losses (blue) is shown across the genome.

(D) Inferred fractions (proportion of positive cells, %) of fibroblasts, $CD8^{+}$ T cells, $CD4^{+}$ T cells, B cells, eosinophils, monocytes, neutrophils, and NK cells across MOC^{K1} (external validation), and MOC^{K2} (external validation), with normal ovary as reference. Statistical comparisons were made using unpaired two-tailed t-test or Mann-Whitney U test, as appropriate. ns, not significant.
